# Design and Analysis of Native Photorespiration Gene Motifs of Promoter Untranslated Region Combinations Under Short Term Abiotic Stress Conditions

**DOI:** 10.3389/fpls.2022.828729

**Published:** 2022-02-16

**Authors:** Debarati Basu, Paul F. South

**Affiliations:** Department of Biological Sciences, Louisiana State University, Baton Rouge, LA, United States

**Keywords:** *cis*-regulatory elements, luciferase activity, high light intensity, high temperature, synthetic biology, photorespiration, gene expression

## Abstract

Quantitative traits are rarely controlled by a single gene, thereby making multi-gene transformation an indispensable component of modern synthetic biology approaches. However, the shortage of unique gene regulatory elements (GREs) for the robust simultaneous expression of multiple nuclear transgenes is a major bottleneck that impedes the engineering of complex pathways in plants. In this study, we compared the transcriptional efficacies of a comprehensive list of well-documented promoter and untranslated region (UTR) sequences side by side. The strength of GREs was examined by a dual-luciferase assay in conjunction with transient expression in tobacco. In addition, we created suites of new GREs with higher transcriptional efficacies by combining the best performing promoter-UTR sequences. We also tested the impact of elevated temperature and high irradiance on the effectiveness of these GREs. While constitutive promoters ensure robust expression of transgenes, they lack spatiotemporal regulations exhibited by native promoters. Here, we present a proof-of-principle study on the characterization of synthetic promoters based on *cis*-regulatory elements of three key photorespiratory genes. This conserved biochemical process normally increases under elevated temperature, low CO_2_, and high irradiance stress conditions and results in ∼25% loss in fixed CO_2_. To select stress-responsive *cis*-regulatory elements involved in photorespiration, we analyzed promoters of two chloroplast transporters (*AtPLGG1* and *AtBASS6*) and a key plastidial enzyme, *AtPGLP* using PlantPAN3.0 and AthaMap. Our results suggest that these motifs play a critical role for *PLGG1*, *BASS6*, and *PGLP* in mediating response to elevated temperature and high-intensity light stress. These findings will not only enable the advancement of metabolic and genetic engineering of photorespiration but will also be instrumental in related synthetic biology approaches.

## Introduction

The construction and expression of multiple genes simultaneously are necessary to engineer complex genetic circuits and regulatory networks underlying almost every metabolic, developmental, and signaling pathway in plants (Lozada et al., 2017; Zhang et al., 2020; Ma et al., 2021). To facilitate such complex multigene engineering, the development of suites of unique gene regulatory elements (GREs) is needed. Early plant engineering attempts relied on repetitive usage of similar GREs, thereby often negatively influencing plasmid stability (Peremarti et al., 2010). Moreover, such approaches have an increased probability of homology-based gene silencing (Oliveira et al., 2010). Stacking genes is a better way of expressing several genes concurrently as the transgenes are most often integrated into nearby regions of the genome, thereby reducing the probability of transgene segregation in the subsequent generations (Halpin, 2005). Two major challenges in plant biotechnology are robust and predictable control of transgene regulation. Constitutive promoters of viral, bacterial or plant origin have played a pivotal role in the robust expression of transgenes and fueled many noteworthy discoveries over the last 40 years. However, most genetic engineering discoveries involve the introduction of single gene vectors or multiple separate gene expression vectors using co-transformation or sequential transformation techniques (Moellenbeck et al., 2001; Manickavasagam et al., 2004; Wu et al., 2014). Increasing evidence has indicated that these traditional strategies suffer from inherent pitfalls due to the complex segregation pattern of transgenes, and random integration of multiple transgenes resulting in wide variation in transgene expressions (Betts et al., 2019). Transforming single vectors containing multiple expression cassettes has emerged as an efficient method for the simultaneous introduction of multiple genes.

Golden Gate assembly is a widely used method for the introduction of multiple genes which offers modular regulatory units and a simple, inexpensive, and rapid way to clone and express multiple genes in plants (Weber et al., 2011; Engler et al., 2014). One of the prerequisites for building multi-gene vectors is the expression of multiple transgenes using unique GREs to avoid co-suppression events (Daniell and Dhingra, 2002). Although numerous constitutive and inducible promoters have been characterized over the last decades, very few studies have analyzed promoter competency of viral, bacterial, and plant-derived promoters within the same experimental setup using multi-gene constructs.

The cauliflower mosaic virus 35S promoter (*35SCaMV*) is one of the most widely used constitutive promoter in plants along with the bacterial *Nopaline synthase (pNOS)* promoter (Amack and Antunes, 2020). The major benefit of using viral or bacterial sequences (*35SCaMV* and *pNOS*) is to obtain strong expression of transgenes in most tissues independent of developmental or environmental constraints (Koncz et al., 1983; Amack and Antunes, 2020). However, a growing number of studies have reported unintended pleiotropic effects in transgenic plants harboring transgenes driven by the *35SCaMV* promoter such as growth retardation, delayed germination, and seed dormancy (Tong et al., 2009; Estrada-Melo et al., 2015). A strong promoter like *35SCaMV* often overloads the regulatory machinery in a targeted cell and leads to gene silencing (Matzke and Matzke, 1995). Additionally, usage of viral and bacterial genetic elements also poses potential biosafety and ethical issues during the future commercialization of transgenic crop plants (Lassen et al., 2002). Such adverse effects are why plant-derived constitutive promoters are favored over viral and bacterial promoters. A number of plant housekeeping genes promoters like ubiquitin (*UBQ*), *ACTIN*, Eukaryotic elongation factor (*EF1* alpha), and *RUBISCO* have been successfully used for constitutive expression of transgenes (McElroy et al., 1990; Park et al., 2010; Feike et al., 2019). Moreover, recent evidence indicated that *AtUBQ10* promoter was more stable and persistent than *CaMV 35S* in Arabidopsis, tobacco and rice (Grefen et al., 2010; Behera et al., 2015; Peramuna et al., 2018; Wang et al., 2021). While few studies have demonstrated that plant-derived promoters display similar efficacies in driving gene expression in both monocot and dicot species, not all promoters behave similarly (Christensen et al., 1992; Behera et al., 2015; Jores et al., 2021). This necessitates comparing the activity of plant promoters derived from both monocots and dicots to be tested within an assay. However, baring studies by Beringer et al. (2017) and Feike et al. (2019), most studies did not compare plant-derived constitutive promoters from various species within a single assay. Besides, plant-derived constitutive promoters, another excellent option to achieve robust and precise expression is the usage of synthetic promoters (Peramuna et al., 2018; Ali and Kim, 2019; Cai et al., 2020; Jores et al., 2021). Since synthetic promoters are assembled linking *cis*-regulatory elements of various promoter regions upstream of the core promoter (TATA box), one can combine tissue specificity with heightened expression within the same promoter construct. Unfortunately, unlike yeast or animal genomics, very few studies have been devoted to identifying core *cis*-regulatory elements underlying developmental and biochemical pathways in plants to help us generate an array of synthetic promoters for gene stacking in crop plants (Kaplan et al., 2006; Kaur et al., 2017; Laxa and Fromm, 2018; Eseverri et al., 2020; Jores et al., 2021; Kakei et al., 2021). In addition to promoters, upstream and downstream untranslated regions (UTRs) also have a profound effect on gene expression (Diamos and Mason, 2018; Yamamoto et al., 2018; de Felippes et al., 2020).

Synthetic biology provides tools to characterize not only the effects of regulatory elements on multi-gene construct design but also provides opportunities to engineer solutions to global challenges in plant productivity. There is a pressing need to increase the photosynthetic efficiency of crops to provide for the world’s increasing population growth as well as higher demands for plant-based biofuels while accounting for the reduction in arable lands due to urbanization (Ort et al., 2015). One method in improving photosynthetic efficiency is to minimize the loss of CO_2_ in the process of photorespiration (South et al., 2018; Batista-Silva et al., 2020). Photorespiration is a multi-organelle process that recycles inhibitory metabolites resulting from Rubisco’s oxygenase activity and is only second to photosynthesis in carbon flux in plant primary metabolism. Furthermore, elevated temperature, drought stress and high light intensities greatly increase photorespiration rates, thereby resulting in an even further reduction in crop yields (Sharkey, 1988; Bauwe et al., 2012; Slattery and Ort, 2019).

Despite the knowledge of the core photorespiratory enzymes very little is known about how they are regulated (Rojas et al., 2012; Saji et al., 2017). Recent reports have shown the potential of utilizing *cis*-regulatory elements (CREs) of endogenous genes to fine-tune transgene expression for preserving spatiotemporal regulatory characteristics (Kaur et al., 2017; Cai et al., 2020; Jores et al., 2021; Kakei et al., 2021; Melo et al., 2021). Synthetic promoters designed with single or multiple CREs identified from native photorespiratory genes can provide valuable insights into the potential positive and negative regulators of photorespiration. The introduction of synthetically designed alternative photorespiratory (PR) bypasses for improving photosynthetic efficiency have attracted a great deal of attention in recent years. Such modified PR pathways have been introduced into Arabidopsis, tobacco and rice; all of which displayed enhanced photosynthetic efficiency and increased biomass (Kebeish et al., 2007; Shen et al., 2019; South et al., 2019).

Here, we assessed the efficiency of an array of viral (*35SCaMV*), bacterial (*pNOS*, *pMAS*), fungal (*WY7*) and plant-derived promoters (*AtUBQ10*, *AtRbcS*, *AtACT2*, *AtSCP30, OsACT2*, *OsEIF5*, *OsCc1*, *OsAPX*, *OsPDG1*, *OsRIGIB*, *OsSCP1*, *OsUBI3, LjUBI*) and UTRs (11- 3′UTRs and 4- 5′UTRs) using a dual-luciferase reporter assay system coupled with transient expression in *Nicotiana benthamiana* leaves. Since, even constitutive expression can be modulated by photoperiod, temperature and the developmental stage of the plants (Obertello et al., 2005; Boyko et al., 2010), we investigated the effect of elevated temperature on constitutive promoters and UTR combinations. In addition, we chose three core photorespiratory genes, plastidic glycolate glycerate transporter (*PLGG1*), 2-PG phosphatase (*PGLP*) and Bile Acid Sodium Symporter 6 (*BASS6*) for studying native CREs that govern their regulation (Schwarte and Bauwe, 2007; Pick et al., 2013; South et al., 2017). *In silico* analysis using PlantPAN 3.0 identified seven different stress-responsive CREs shared by all three genes (Chow et al., 2016). Adopting a synthetic promoter design strategy, we generated synthetic promoters by using repeats of the identified CREs followed by a core promoter region (TATA box) (Ali and Kim, 2019). Using tobacco transient expression in conjunction with a dual-luciferase reporter assay we have characterized both constitutive and three photorespiration promoter elements in response to elevated temperature, low CO_2_ and high light intensity. These findings indicate the potential positive and negative regulators of key plastidial photorespiratory genes in response to prominent abiotic stressors. Collectively, these findings will help in predictably expressing transgenes for genetic engineering of metabolic processes in plants under controlled and stress conditions.

## Materials and Methods

### Plant Materials and Growth Conditions

*Nicotiana benthamiana* seeds were planted in Sungrow mix soil and grown in 12 h day/night cycles in a Percival growth chamber with light intensity set for 150 μmol photons m^–2^ s^–1^ at a temperature of 22°C for day temperature and 20°C night temperature with relative humidity set at 50%. Plants used in this study were between 4 and 6 weeks old. Ambient growth condition involves ∼400 ppm of CO_2_, 150 μmol photons m^–2^ s^–1^ light intensity, and 22°C of temperature, whereas elevated temperature denotes 32°C and high light intensity denotes 1200 μmol photons m^–2^ s^–1^. For experiments dealing with high light intensity stress has a temperature regime of 25°C. The low CO_2_ levels were generated by passing air through soda lime and CO_2_ levels were monitored using a sensor.

### Cloning

The GoldenGate modular cloning system was used for cloning and assembly of desired constructs using standardized overhangs (Weber et al., 2011; Patron et al., 2015). Level 0 (L0) gene fragments or circular plasmids (promoters, UTRs, coding regions) were synthesized either in collaboration with the Engineering Nitrogen Symbiosis for Africa ENSA group (University of Cambridge, Cambridge, United Kingdom) or Twist Biosciences (San Francisco, CA, United States), containing defined Golden Gate compatible overhangs. In the case of linear fragments, traditional digestion followed by ligation was performed with Golden Gate compatible acceptors. Next, L1 and L2 plasmids harboring gene cassettes were assembled by combining L0 modules into expression units containing the promoter- UTR (PU) coding sequence (SC) and terminator (T) modules using type IIs restriction enzyme *Bsa*I-HF and T4 ligase (New England Biolabs, Beverly, MA, United States). Finally, combining L1 modules, level 2 plasmids containing multiple gene expression cassettes were constructed using type IIs restriction enzyme *Bbs*I-HF and T4 ligase (New England Biolabs). All L0, L1 and L2 plasmids constructs synthesized for this paper are confirmed by diagnostic digestion followed by sequencing the end-junctions of the inserts. The details of the L0 modules, L1 and L2 plasmids used in this study are listed in Supplementary Table S1. Acronyms of various GREs are as follows Ω*TMV*, 5′UTR from tobacco mosaic virus; MP, minimal promoter devoid of any CREs; 1X*p35SCE*, single CRE of C*aMV*; *pCaMV35SFL* or *p35SFL*, full length C*aMV* promoter; *pNOS*, *Agrobacterium Nopaline Synthase*; *pWY7, Oidium heveae* promoter; *pMASPU, Mannopine Synthase*; *pUBQ10PU*, *Arabidopsis thaliana ubiquitin-10; pAtACT2, Arabidopsis thaliana ACTIN 2; pOsPGD1, Oryza sativa phosphogluconate dehydrogenase; pOsEIF5, Oryza sativa Initiation factor; pOsAPX2, Oryza sativa ascorbic peroxidase; pOsCc1, Oryza sativa cytochrome-c; pOsR1G1B, Oryza sativa R1G1-domain-containing protein; LjUBI, Lotus japonicus polyubiquitin gene; pAtSCPL30, Arabidopsis thaliana Serine carboxy peptidase-like; pAtRbcS, Arabidopsis thaliana ribulose-1,5-bisphosphate carboxylase/oxygenase small subunit. 5*′*ADH*, alcohol dehydrogenase; *5*′*AGP21*, Arabinogalactan 21; 5′*CAB22L*, chlorophyll a/b binding gene from petunia; *CaMV35ST or 35ST*, C*aMV terminator; NOST, Agrobacterium Nopaline Synthase; OCST; Octopine synthase; MAST*, *Mannopine Synthase;* HSP, heat shock protein; Ag7T, Agrobacterium-derived Ag7 terminator, CPMV, Cowpea mosaic virus; RbcST, *Arabidopsis thaliana ribulose-1,5-bisphosphate carboxylase/oxygenase small subunit* terminator; *ACT2T*, *Arabidopsis thaliana ACT2* terminator.

### *In silico*-Analysis of Promoter Regions

To examine and identify the CREs in the promoter region of the core PR genes At*PGLP*, At*BASS6* and At*PLGG1* a two independent web-based promoter analysis tools, AthaMap^1^ (Hehl et al., 2016) and PlantPAN 3.0^2^ (Chow et al., 2019) were used. Briefly, 2-kb upstream regions of the predicted start codon (ATG) were submitted. The common transcription factor binding sites were identified by selecting the co-occurrence analysis using the ‘Gene group analysis’ function of PlantPAN 3.0 with the following settings: the threshold for co-occurrence support was kept at >90% confidence and distance constraint at 100 bp. We selected seven different classes of stress-responsive CREs each with four different motifs for further analysis.

### *Agrobacterium tumefaciens* Transformation

*Agrobacterium tumefaciens* strain GV3101 was transformed with 500 ng of each plasmid by electroporation. Transformants were rescued in 1 ml LB with shaking at 28°C for 1.5–2 h and plated onto LB agar containing 50 μg/μl carbenicillin and 50 μg/μl gentamycin. A single colony from each construct was inoculated after 2 days of growth at 28°C.

### *Agrobacterium*-Mediated Transient Expression

*Agrobacterium tumefaciens* strain GV3101 transformed with each construct was grown at 28°C overnight in 5 ml of LB medium supplemented with appropriate antibiotics. Transformed Agrobacterium cells were resuspended in 1 ml of *Agrobacterium* Infiltration Medium (10 mm MES pH 5.8, 10 mm MgCl_2_ with 150 μM acetosyringone) and incubated for 2 h at 28°C. Each target strain was maintained at and an optical density of OD_600_ = 0.5 per strain and was co-infiltrated with OD_600_ = 0.3 Nano-LUC plasmid and OD_600_ = 0.3 of *p19* gene from Tomato bushy stunt virus for suppression endogenous silencing. Leaves of 4- to 6-week-old plants were then infiltrated with the culture mixture, and the plants were grown for 3 days after infiltration before leaf disks were recovered for analysis. In case of elevated temperature, high light intensity treatments, or low CO_2_ treatment, infiltrated tobacco plants were subjected to the stress treatment conditions 2 days after infiltration for indicated timespan.

### Luciferase Image Analysis

Imaging was performed as described in Yu et al. (2007) with minor modification. Infiltrated tobacco leaves were left for 3 days, the abaxial sides of the infiltrated leaves were sprayed with luciferin (1 mM luciferin and 0.01% Triton X-100) and then kept in the dark for 10 min. Luminescence was also visualized using a CCD camera (ChemiDoc XRS; Bio-Rad, Hercules, CA, United States) with 2 × 2 binning settings for all images. Images were acquired with a 5-min exposure time followed by quantifying the luminescence intensity of each leaf by the integrated density method in ImageJ.

### Promoter Activity Assay

Leaf disks from infiltrated leaves were assayed using the Dual-Luciferase Reporter Assay System (Promega, E1910, Madison, WI, United States), following the manufacturer’s instructions. Three independent trials, each with three biological replicates, were performed for each construct. Briefly, 3–4 leaf disks per infiltrated leaves were homogenized in 150 μL of extraction buffer (20 mm Tris-HCl, pH 7.5, 150 mM NaCl, 5 mM MgCl_2_, 5 mM DTT). Twenty microliters of crude extract were mixed with 75 μL of LUC assay buffer, and the LUC activity was monitored spectrometrically using a plate reader (Synergy LX multi-mode plate reader; Biotek). Next, 75 μL of Stop and Glow buffer was added for the measurement of the *Renilla* luciferase activity. For the final transcriptional activity, the firefly luciferase and *Renilla* luciferase were measured sequentially, and the Fluc:Rluc ratio was calculated. Normalized data are presented as the ratio of the luminescent signal intensity for reporter versus internal control reporter (*pNOS*) from three independent biological samples.

### RNA Isolation and Quantitative Real-Time Polymerase Chain Reaction (qPCR) Analysis

Total RNA was extracted from 3-week-old leaves of *Nicotiana*. *benthamiana* using the RNA Plus Kit (Macherey-Nagel, Allentown, PA, United States) following manufacturer’s instructions. gDNA contaminations were removed with the NucleoSpin gDNA removal columns included in the kit. Next, equal amounts of RNA samples from each time point and treatment (1000 ng) were reverse transcribed to synthesize cDNA was using QuantiNova Reverse Transcription Kit (Qiagen, Germantown, MD, United States) according to the manufacturer’s instructions. Finally, quantitative real-time polymerase chain reaction (qPCR) analysis was performed on a QuantStudio 3 real-time PCR instrument (Thermo Fisher, Waltham, MA, United States) using PowerUp SYBR Green Master Mix (Applied Biosystems, Foster City, CA, United States) with primer pairs specific to genes of interest, along with the reference genes. *NbL23* and *NbEF1*α genes were used as an internal control, a geometric mean of the two reference genes was used for normalizing expression levels of *NbPLGG1*, *NbBASS6*, *NbPGLP* genes (Liu et al., 2012). Relative transcript levels were measured in three independent experiments and each reaction with three technical triplicates using the ddCt method (Livak and Schmittgen, 2001). The following thermoprofile: 50°C 2 min, 95°C 10 min followed by 40 cycles of 95°C for 15 s and 60°C for 1 min and 95°C for 1 s was opted. All qPCR primers are listed (Supplementary Table S2).

### Statistical Analysis

Statistical analysis and graphing were performed with GraphPad Prism version 7.04. The error bars presented in the figures are standard deviations (±sd). In all cases, statistically significant differences were considered at *p* < 0.05. Statistical significance was determined by Welch’s unpaired *t*-test (two groups), one-way ANOVA (more than two groups) or two-way ANOVA (more than two groups with two variables). All ANOVA analyses were followed by Turkey’s multiple comparisons.

### Accession Numbers

Sequence data from this article can be found in the GenBank/EMBL data libraries under the following accession numbers:

*AtAct2 P (LR782544.1), AtUBQ (LR782545), AtRbcS1B (NM_123203) OsAPX (AK068430), OsSCP1 (AK101133), OsPGD1 (AK065920), OsR1G1B (AF503583), OsEIF5 (AK060387), OsCc1 (AK060267), OsAct1 (AK100267), LjUBI1 (AP009383.1), NbEF1*α *(AY206004), NbL23 (XM_016579891), PsRbcST- (JN903571.1), AtHSP18.2 (NM_125364.3), AtADH (AY536888), AtH4T (M17132.1) AtAct2 T (U41998.1), AtAGP21 (NM_104409.3)*, *AtSCP30L (LR782545.1)*, *Oidium heveae-WY7 (MN889519.1), Petunia* gene for chlorophyll a/b binding protein cab 22L (*X02359.1*).

## Results

### Evaluation of Diverse Promoters and Untranslated Regions on Luciferase Activity

Of the numerous factors that can influence transgene gene expression, promoters are perhaps the most important. We developed a rapid screening method to test the strength and efficacies of several promoters and UTRs by employing a dual luciferase-based transient transcriptional activity assay in *Nicotiana benthamiana*. A ratiometric dual-luciferase assay was selected to account for the variation in transformation events between agroinfiltrations (Solberg and Krauss, 2013). Furthermore, we have incorporated another step, *in vivo* luminescence imaging, to ensure reliable luciferase activity among replicates. To validate reproducibility and robustness of our transient reporter assay, agroinfiltrated leaves harboring luciferase gene under the control of well-characterized constitutive promoters were imaged for luminescence after 2-day post infiltration. As expected, *p35SFL:LUC* showed strong luciferase activity followed by *pAtUBQ10-PU:LUC* and *pAtRbcS:LUC* (Supplementary Figures S1A,B). A strong correlation was observed between *in vivo* and *in vitro* luciferase activity upon testing the same constitutive promoters (Supplementary Figures S1B, S2A). To capture maximum luciferase activity, we performed a time-resolved luminescence assay by collecting infiltrated leaf samples at indicated days post infiltration. Monitoring time-course luciferase activity indicates that 48–72 h post agroinfiltration is ideal for assessing promoter and UTR efficacies. As shown in Supplementary Figure S2B, the activity of the *pAtUBQ10-PU* promoter showed a peak at 48 h that lasts past 72 h (∼6-fold) followed by a gradual decline at 84 h (∼4.8-fold).

**FIGURE 1:**
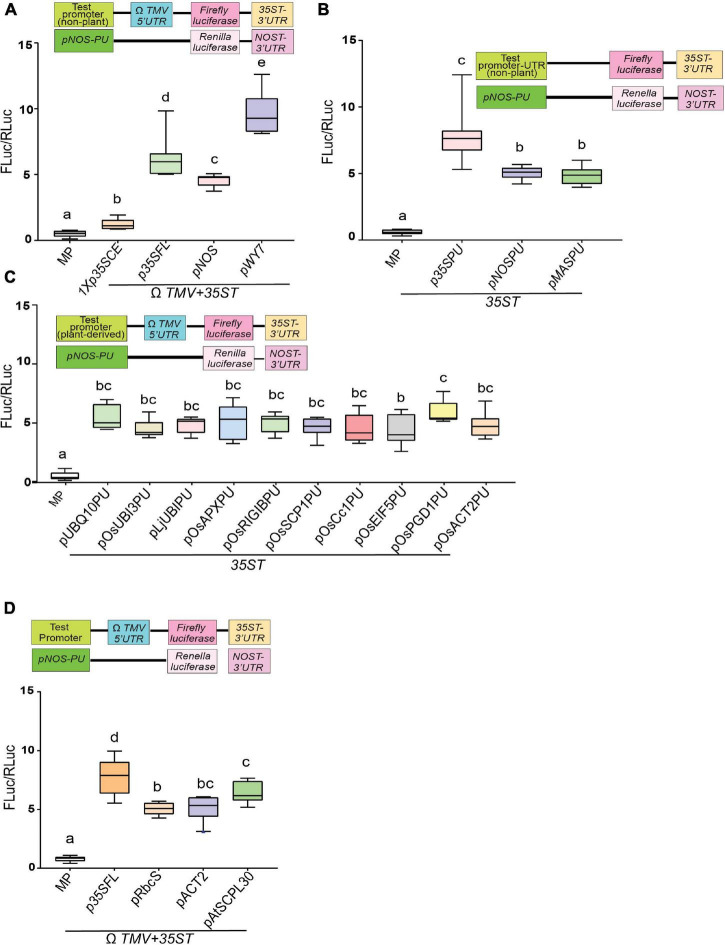
Efficacy comparison of diverse constitutive promoters using a transient dual luciferase reporter assay. **(A)** Strength of viral, bacterial and fungal constitutive promoters as assessed by quantitative luciferase activity. **(B)** Strength of viral and bacterial promoter-5′UTR combinations as assessed by quantitative luciferase activity. **(C)** Strength of diverse plant-derived promoters-5′UTR combinations as assessed by quantitative luciferase activity**. (D)** Strength of three Arabidopsis constitutive promoters compared to the *p35SFL* as assessed by quantitative luciferase activity. Luciferase activity within the infiltrated tobacco epidermal cells was quantified by measuring light emission in a luminometer. Promoter activity is expressed as the relative activity of firefly luciferase versus *Renilla* luciferase (Fluc/Rluc). Box-whiskers plots represent data from three independent experiments, each with three biological replicates. Letters indicate statistical significance based on a one-way ANOVA with Tukey’s multiple component analysis (*p* < 0.05); samples sharing letters are not significantly different. Error bar represents a mean ± standard deviation. The UTRs used are indicated by the line at the bottom. The insets in all panels represent schematic representation of constructs used for testing efficacies of constitutive promoters used in this study. Acronyms used are defined in the ‘Materials and Methods’ section.

We systematically evaluated the ability of several constitutive promoters and UTRs of various origins in gene expression strength using transient quantitative luciferase assay as illustrated in Supplementary Figure S2C. We have divided promoters into the following categories – (i) non-plant promoter, (ii) promoter-5′UTR combinations (PU), (iii) plant-derived promoters-5′UTR combinations (PU), and (iv) plant-derived promoter. In the case of discrete promoter sequences, 5′UTR from the genomic RNA of tobacco mosaic virus, known as the omega leader (5′-UTR-Ω*TMV*), was incorporated. Among non-plant promoters, fungal promoter *pWY7* (Wang et al., 2020) displayed the highest luciferase activity (∼9.7-fold), followed by *p35SFL* (∼6.8-fold) (Odell et al., 1985) and *pNOS* (∼5-fold) (Sanders et al., 1987; Figure 1A). As expected, barely any luciferase activity was detected for minimal promoter (MP), which contains no activation elements upstream of the TATA motif. In contrast, 1X*p35SCE* (∼1.9-fold) displayed a slightly higher activity compared to *MP* (∼0.97-fold), as it contains a CRE activation element from the *CaMV35S* gene, upstream of the TATA motif (Cai et al., 2020). Next, we tested non-plant promoters fused to their native 5′UTRs to compare upstream gene regulatory elements effectively. As shown in Figure 1B, the viral promoter-5′UTR combinations (*p35SPU*; ∼7-fold) drove the highest luciferase activity followed by two bacterial promoters *pNOSPU* (∼5.1-fold) and *pMASPU* (∼4.9-fold) (De Bolle et al., 2003).

Very few studies have compared dicot and monocot promoters side-by-side (Feike et al., 2019). This led us to examine diverse promoter-5′UTR combinations, including *pAtUBQ10PU*, *pOsUBI3PU*, *pOsEIF5PU, pOsAPXPU*, *pOsPGD1PU*, *pOsRIGIBPU*, *pOsSCP1PU*, *pOsCc1PU*, *pOsACT2PU*, *LjUBIPU*. All plant-derived promoters led to detectable luciferase activity to a similar extent (4.7- to 5.3-fold), regardless of the species of origin (Figure 1C). Finally, among promoters derived from Arabidopsis, *pAtSCPL30* (∼6.5-fold) (An et al., 1996; Jiang et al., 2018) exhibited the highest luciferase activity followed by *pAtACT2* (∼5.2-fold) and *pAtRbcS* (∼5.1-fold) (Martìnez-Hernaàndez et al., 2002; Figure 1D). However, as expected, none of the plant-derived promoters displayed luciferase activity comparable to *p35SFL* (∼8.5-fold) (Feike et al., 2019). Considered together, our study demonstrates that among non-plant promoter’s the fungal promoter (*pWY7)* displayed the highest luciferase activity (∼20% higher than *p35SFL*). Furthermore, plant-derived promoters obtained from dicots, legumes, or monocots have similar luciferase activity.

### Analysis of 5′ and 3′ Untranslated Region Combinations on Luciferase Activity

**FIGURE 2:**
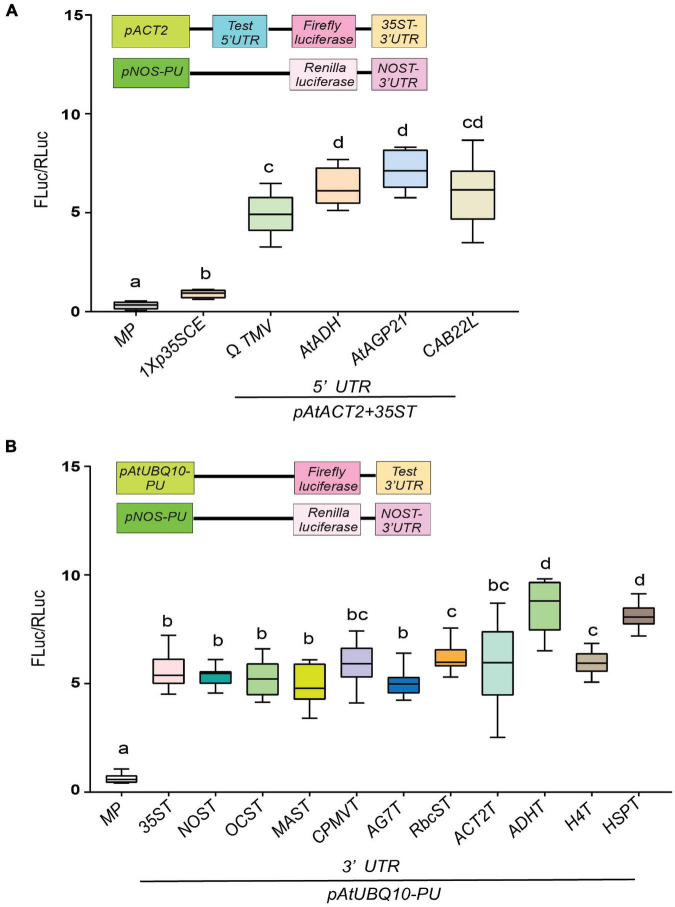
Evaluation of the effect of diverse untranslated (UTR) gene elements on translation as assessed by transient dual luciferase reporter assay. **(A)** Comparison of four diverse 5′UTR elements. **(B)** Comparison of 11 diverse 3′UTR elements. All 5′UTR elements containing gene cassettes comprises of *pAtACT2*; *Fluc: 35ST* whereas, all the 3′UTR containing gene cassettes comprised of *pAtUBQ10*; Ω*TMV; Fluc.* The common promoters and UTRs used are indicated by the line at the bottom. Data analyzed as described in Figure 1. Box-whiskers plots represent data from three independent experiments, each with three biological replicates. Letters in the indicate statistical significance based on a one-way ANOVA with Tukey’s multiple component analysis (*p* < 0.05); samples sharing letters are not significantly different. Error bar represents a mean ± standard deviation. The insets in all panels represent schematic representation of constructs used for testing efficacies of UTRs used in this study. Acronyms used are defined in the ‘Materials and Methods’ section.

While promoters are often considered the most predominant regulatory element of gene expression by recruiting transcription factors, untranslated regions at the 5′ and 3′ regions of a gene play a critical role in regulating gene expression (Srivastava et al., 2018). However, the influence of UTRs on gene expression has not been investigated to its fullest potential. Toward this goal, we have generated constructs to evaluate the effect of several non-plant and plant-derived 5′ and 3′ UTRs on luciferase activity. The 5′UTR plays a prominent role in enhancing mRNA translation (Satoh et al., 2004; Yamasaki et al., 2018a,b). The most widely used 5′UTR in plant gene expression are viral leader sequences (Gallie and Walbot, 1992; Fan et al., 2012). Here, we have tested the efficacies of well-characterized non-plant (Ω*TMV* 5′UTR) and plant-derived UTRs namely, *AtAGP21*, *AtADH* and *CAB22L* 5′UTR side by side (Satoh et al., 2004; Wever et al., 2010; Matsui et al., 2012). As shown in Figure 2A, two plant-derived 5′UTRs, *AtAGP21* (∼5.8-fold) and *AtADH* (∼5.2-fold) resulted in higher luciferase activity compared to viral Ω*TMV* 5′UTR (∼4.9-fold). Additionally, *CAB22L* 5′UTR (∼5-fold) derived from petunia exhibited similar luciferase activity as that of Ω*TMV* 5′UTR. As expected, the lowest luciferase activity was detected in 1X*p35SCE* (∼1.9-fold) followed by *MP* (∼0.91-fold).

Like 5′UTRs, the 3′UTR also plays important role in regulating gene expression by controlling precise transcriptional termination and mRNA processing (Bernardes and Menossi, 2020). This led us to investigate the efficacies of 11 different 3′UTRs of various origins. Two 3′UTRs, namely *ADHT* (9.1-fold) and *HSPT* (∼8.8-fold) showed the highest luciferase activity followed by *H4T* (∼7-fold) and *RbcST* (∼6.8-fold) (Nagaya et al., 2010; Figure 2B). As expected, the lowest luciferase activity was detected in *MP* (∼0.98-fold).

Taken together, our results indicate that 5′UTR *AtADH* and *AtAGP21* and 3′UTR *HSPT* and *ADHT* displayed enhanced luciferase activity (∼9–12% higher) among the corresponding UTRs tested (Figure 2).

### Effect of Elevated Temperature and High Light Intensity on the Efficacy of Selected Constitutive Promoter and Untranslated Region Combinations

Amongst all the abiotic stresses that adversely affect plant yield in fields, elevated temperature stress condition has gathered immense attention due to concerns over climate change effects on plant growth. A number of studies have attributed global yield losses in key crop plants to increases in temperature during growing seasons (Hatfield and Prueger, 2015; Zhao et al., 2017; Agnolucci et al., 2020; Moore et al., 2021). Thus, transgenic plants that are grown in greenhouse or field conditions may experience elevated temperature stress. To get a better understanding of commonly used constitutive promoters under abiotic stress conditions we tested the expression strength of selected constitutive promoters and UTR combinations in response to elevated temperature and high light intensity. Two days post agroinfiltration, transformed leaves with indicated promoters-UTR combinations were shifted to elevated temperature (32°C) for 3 h. All the selected constitutive promoters exhibited expression trends of luciferase activity under elevated temperature conditions as they were comparable to expression under ambient controlled conditions (25°C) (Supplementary Figures S3A,B). Likewise, selected 5′ and 3′UTR also did not display any discernable differences in their strength to drive luciferase activity in response to elevated temperature compared to ambient conditions (Supplementary Figures S3C,D. Overall, our results revealed that luciferase activity driven by the selected constitutive promoters and UTRs remain unaffected by elevated temperature stress at 32°C for a duration of 3 h.

### Assessment of New Combinations of Constitutive Promoters and Untranslated Regions

**FIGURE 3:**
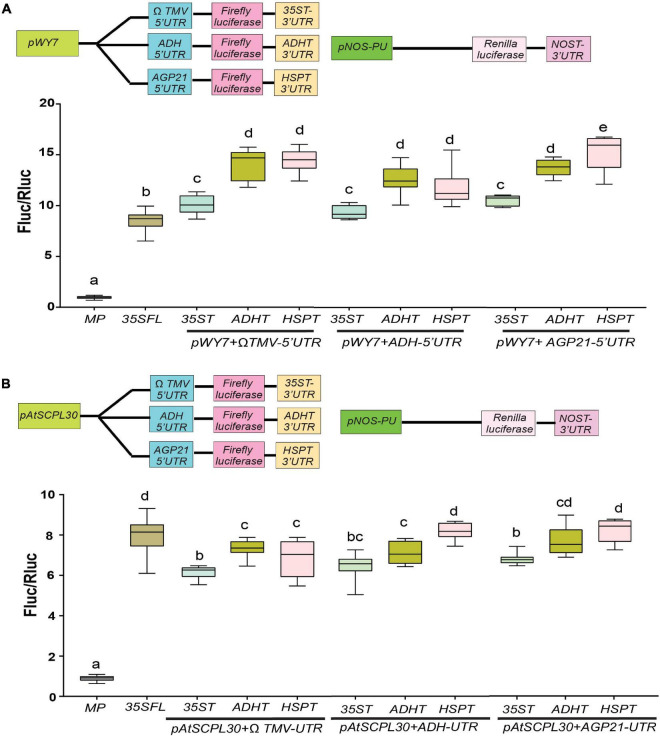
Evaluation of efficacies of selected constitutive promoter-UTR combinations. **(A)** Relative luciferase activity driven by a fungal constitutive promoter (pWY7) with diverse UTR combinations. **(B)** Relative luciferase activity driven by a plant-derived constitutive promoter (AtSCPL30) with diverse UTR combinations. The common promoters and UTRs used are indicated by the line at the bottom. Data analyzed as described in Figure 1. The insets in all panels represent schematic representation of constructs used for testing efficacies of constitutive promoters-UTR combinations used in this study. Box-whiskers plots represent data from three independent experiments, each with three biological replicates. Error bar represents a mean ± standard deviation. Letters indicate statistical significance based on a one-way ANOVA with Tukey’s multiple component analysis (*p* < 0.05); samples sharing letters are not significantly different. Acronyms used are defined in the ‘Materials and Methods’ section.

Based on our results in Figures 1, 2, we hypothesize that an enhanced gene expression can be achieved if we select and combine the best performing isolated promoters and (*pWY7* and *AtSCPL30*), 5′UTRs (*AtADH* and *AtAGP21*) and 3′UTRs (*ADHT* and *HSPT*). This led us to construct nine different gene expression cassettes to test their combined effects. For effective comparison, we compared luciferase activity driven by *p35SFL:*Ω*TMV: 35ST* as a control. The *pWY7* promoter with plant-derived 5′UTRs (*AtADH* and *AtAGP21*) or 3′UTRs (*ADHT* and *HSPT*) displayed comparable luciferase activity compared to *pWY7* with viral UTRs, Ω*TMV* and *35ST* (Figure 3A). Next, we investigated the efficacy of plant promoter *pAtSCPL30* linked with plant-derived UTRs. Like *pWY7*, plant promoter *pAtSCPL30*, also showed similar luciferase activity when combined with either of the two 5′UTRs or 3′UTRs in comparison to the viral UTRs (Figure 3B). However, consistent with our previous findings, *pWY7* and *p35SFL* displayed higher luciferase activity (∼20–35%) than *AtSCPL30*, irrespective of UTR combinations. Collectively, plant-derived UTRs showed comparable luciferase activity when linked to either fungal *pWY7* or plant *pAtSCPL30* promoters, compared to viral UTRs. This provides an opportunity to replace viral UTRs with more desirable plant-derived UTRs.

To examine the promoter-UTR combinations under abiotic stress conditions, 2-day post agroinfiltration, transformed leaves with indicated promoters-UTR combination were shifted to either elevated temperature (32°C) for indicated time-points or high light intensity (1200 μmol photons m^–2^ s^–1^) for 3 h. Consistent with previous findings, under ambient conditions (25°C; 150 μmol photons m^–2^ s^–1)^ *pWY7* promoter with plant-derived UTRs displayed comparable luciferase activity compared to viral UTRs (Supplementary Figure S4A). When subjected to elevated temperature, only *pWY7*E (5′UTR-*ADH-ADHT*), exhibited higher luciferase activity (∼4%) among all different combinations compared to ambient conditions. Unlike *pWY7*E, a reduced luciferase activity (∼5%) was displayed in *pWY7*G (5′UTR-*AGP21-HSPT*) combinations, compared to ambient conditions (Supplementary Figure S4A). Contrary to luciferase activity in response to elevated temperature, upon high light intensity stress, only *pWY7*D (5′UTR-*AGP21-HSPT*), displayed higher luciferase activity (∼7%) compared to ambient conditions (Supplementary Figure S4A). Similar to elevated temperature response, *pWY7*G displayed reduced luciferase activity (∼5%) in response to high light stress. Next, we examined plant promoter, *AtSCPL30* (*pS*) with various combinations of UTRs. In response to elevated temperature, only *pSC* (Ω*TMV-35ST)* and *pScD* (5′UTR-*AGP21-HSPT*) displayed reduced luciferase activity (∼5%) compared to ambient conditions (Supplementary Figure S4B). Upon high light intensity stress, a reduced luciferase activity (∼5%) was observed in *pSD* (5′UTR-*AGP21-HSPT*) combinations, whereas *pS*A (Ω*TMV-HSPT)* displayed an increase in luciferase activity (∼7%)- compared to ambient conditions. Neither elevated temperature nor high light intensity stress conditions have any noticeable change on luciferase activity driven by the *35SFL* promoter. Taken together, plant UTRs seemed to be more sensitive toward high temperature and high light stresses compared to viral UTRs.

### Effect of Elevated Temperature, High Light Intensity, and Low CO_2_ on Expression of Selected Photorespiratory Genes

Since overexpression of a transgene driven by constitutive promoters often results in unintended pleiotropic effects, there has been a considerable drive to study synthetic promoters (Kaur et al., 2017; Ali and Kim, 2019; Cai et al., 2020; Jores et al., 2021). Synthetic promoters are typically chimeric, they are built with TATA box sequence, upstream single or multiple identical or different CREs obtained from selected non-native or native promoters, and intervening spacer sequences. As a proof-of-principle, we have chosen to identify and characterize *cis*-regulatory elements using genes involved in photorespiration. Photorespiration is a highly conserved metabolic process that lies at the crossroads of primary metabolism, environmental stress response and crop improvement. Although, considerable progress has been made to biochemically characterize the genes involved in photorespiration the mechanism of regulation of these genes in response to unavoidable environmental cues is at its inception (Laxa and Fromm, 2018). A number of recent reports have suggested that key photorespiratory genes, including *PLGG1*, *GOX1*, *PGLP1*, *HPR1* are differentially regulated by elevated temperature and high light intensity (Song et al., 2014; Balfagón et al., 2019; Timm et al., 2019; Anderson et al., 2021; Moore et al., 2021).

**FIGURE 4:**
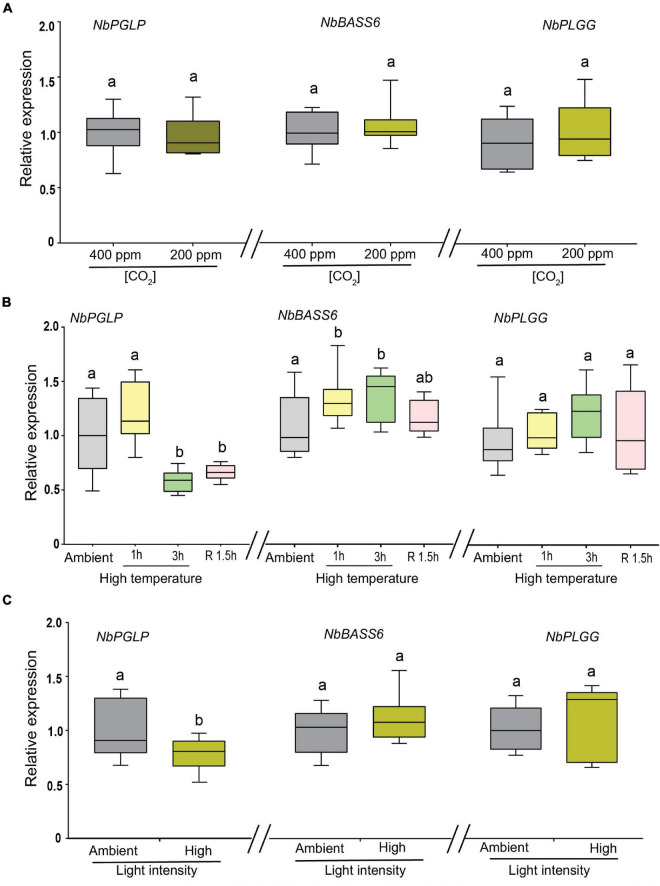
Expression of *NbPGLP*, *NbBASS6* and *NbPLGG* in response to low CO_2_, elevated temperature and high light intensity. **(A)** Plants were grown under continuous illumination with 400 ppm of CO_2_ before shifting them to low CO_2_ (200 ppm) for 24 h. **(B)** Plants were subjected to elevated temperature at 32°C for indicated timepoints. Post 3 h high temperature stress, plants were shifted back to ambient temperature for recovery for 1.5 h (R1.5h). Plants maintained in ambient temperature (22°C) were included for comparison and denoted (ambient). **(C)** Plants were subjected to high light intensity 1200 in μmol/m^–2^s^–1^ for 3 h. Control plants were maintained at 120 in μmol/m^–2^s^–1^. Box-whiskers plots represent relative expression values that were normalized to *NbEF1*α and *NbL23* reference genes and their ratios relative to those of unstressed plants. Data presented are representative of three independent experiments and error bar represents a mean ± standard deviation. Letters indicate statistical significance based on a **(B)** one-way ANOVA followed by Tukey’s multiple component analysis (*p* < 0.05) **(A,C)** Welch’s *t*-test (*p* < 0.05); samples sharing letters are not significantly different.

Recently, Balfagón et al. (2019) reported that both *AtPLGG1* and *AtPGLP1* are differentially expressed in response to heat stress or high light stress, albeit to varying degrees. Herein, we analyzed whether tobacco orthologs are also differentially regulated in response to low CO_2_, elevated temperature and high light intensity. Since many of the photorespiratory mutants have been identified or characterized under low CO_2_ or in absence of CO_2_, we first examined the expression of above mentioned three genes in response to low CO_2_ (200 ppm) compared to ambient CO_2_ conditions (400 ppm). None of these genes exhibited any noticeable change in the transcript levels when subjected to low CO_2_ for 24 h compared to ambient conditions (Figure 4A).

Next, we turned our attention to the impact of high temperature stress on the expression of these three genes. In response to elevated temperature for 1 h, we observed a slight increase in transcript abundance of *NbPGLP*, although it was not statistically significant (Figure 4B; left panel). In contrast, when subjected to an elevated temperature (32°C) for 3 h, a moderate reduction in transcript levels of *NbPGLP* (∼40%) was observed compared to ambient conditions (22°C) (Figure 4B; left panel). Our findings are corroborated by the data from the publicly available transcriptome database (eFP browser) which *AtPGLP* seems to be specifically reduced in response to high temperature (Winter et al., 2007; Supplementary Figure S5A). The reduction in *NbPGLP* transcript levels did not recover after shifting the plants in ambient conditions for 1.5 h. Conversely, *NbBASS6* transcript levels were slightly upregulated (∼10–15%) in response to elevated temperature stress both at 1 h and 3 h compared to ambient conditions (Figure 4B; middle panel). Interestingly, the increase in transcript levels of *NbBASS6* reverts to wild-type levels during the recovery period. Unlike *NbPGLP* and *NbBASS6*, *NbPLGG1* expression levels remain unchanged compared to control under all-time points (Figure 4B; right panel).

When subjected to high light intensity stress (1200 μmol photons m^–2^ s^–1^) for 3 h, reduced transcript levels (∼30%) of *NbPGLP* were observed compared to ambient conditions (150 μmol photons m^–2^ s^–1^) (Figure 4C; left panel). In contrast, neither *NbBASS6* (Figure 4C; middle panel) nor *NbPLGG1* (Figure 4C; right panel) genes displayed any discernable changes in their transcript levels when subjected to high light intensity.

Taken together, our results indicate that *NbPGLP* is downregulated in response to both elevated temperature and high light intensity, even though photorespiration rates increase in both conditions.

### Efficacy Assessment of Synthetic Promoters Containing Native Stress-Responsive *Cis*-Regulatory Elements Derived From Selected Photorespiratory Genes

**FIGURE 5:**
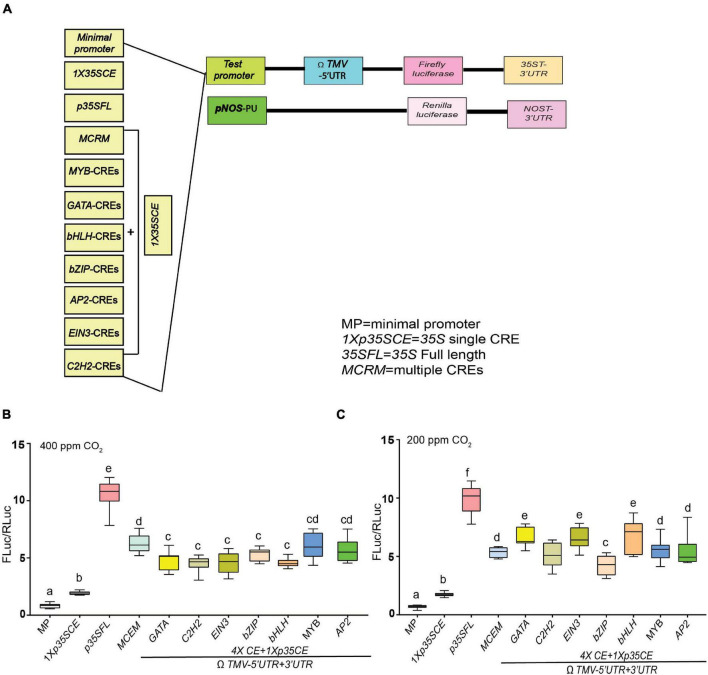
Effect of low CO_2_ on the efficacies of synthetic promoters derived from CREs of core photorespiratory genes. **(A)** Seven different *CREs* were identified by submitting predicted promoter regions of *AtPGLP*, *AtPLGG1* and *AtBASS6* genes and using AthaMap and PlantPAN 3.0. As shown in the illustration, individual *CREs* are linked with short non-functional linker sequences along with proximally placed *1* × *35SCE* promoter for luciferase-based promoter-efficiency assay. Acronyms used are defined in the ‘Materials and Methods’ section and details of the sequence information is listed in Supplementary Table S1. **(B)** Relative luciferase activity driven by synthetic promoters under ambient condition (400 ppm CO_2_). **(C)** Relative luciferase activity driven by synthetic promoters under low CO_2_ (200 ppm CO_2_). Constructs were agroinfiltrated and 2-day post infiltration one set of transformed plants were shifted to a growth chamber with low CO_2_ (200 ppm) for 24 h and control plants were kept at 400 ppm of CO_2_. Data analyzed as described in Figure 1. Box-whiskers plots represent data from three independent experiments, each with three biological replicates. Error bar represents a mean ± standard deviation. Letters indicate statistical significance determined by one-way ANOVA with Tukey’s multiple component analysis (*p* < 0.05); samples sharing letters are not significantly different. The UTRs used are indicated by the line at the bottom. Acronyms used are defined in the ‘Materials and Methods’ section.

To gain insight into the regulation of the key stress-associated photorespiratory (PR) genes, we performed an *in silico* analysis using two web-based promoter analysis tools, AthaMap (see text footnote 1; Steffens et al., 2005) and PlantPAN 3.0 (see text footnote 2; Chow et al., 2019). Next, we selected a functionally diverse class of PR genes, namely *AtPLGG1* and *AtBASS6* (transporters), and *AtPGLP1*, an essential PR enzyme for further analysis. Our analysis selected seven different CREs each offer binding sites for seven well-characterized classes of transcription factors. To test the function of these seven putative stresses- responsive CREs, we created synthetic promoters containing four copies of individual CREs along with a single copy of *35SCRE* (*1Xp35SCE*) followed by a TATA motif fused to a luciferase reporter gene (Figure 5A). For a thorough comparison of the strength of these CREs, a multiple CRE (*MCRM*) comprising all the seven different CREs was included as well as a construct with *1Xp35SCE* and MP. It is widely accepted that photorespiration is stimulated under elevated temperature, low CO_2_ and can be modulated under high light intensity (Huang et al., 2015). This prompted us to investigate whether any of these stress-responsive CREs are responsible for influencing luciferase activity in response to high temperature, high light intensity, or low CO_2_.

In response to ambient CO_2_ (400 ppm), *MCRM* (∼8-fold), followed by *MYB-CRE* (∼7-fold) exhibited higher luciferase activity, compared to synthetic promoters with single CRE (Figure 5B). Interestingly, in comparison to ambient conditions, a modest increase in luciferase activity (∼5–10%) was observed in *GATA-CRE*, *EIN3-CRE*, *bHLH-CRE* in response to low CO_2_ (200 ppm) (Figure 5C). In contrast, in response to low CO_2_, *MCRM* (∼20%) and *bZIP*-CRE (∼5%) displayed a reduction in luciferase activity, compared to ambient conditions (400 ppm). The highest luciferase activity was observed in *p35SFL* (∼10- to 12-fold) regardless of the time points treatments.

**FIGURE 6:**
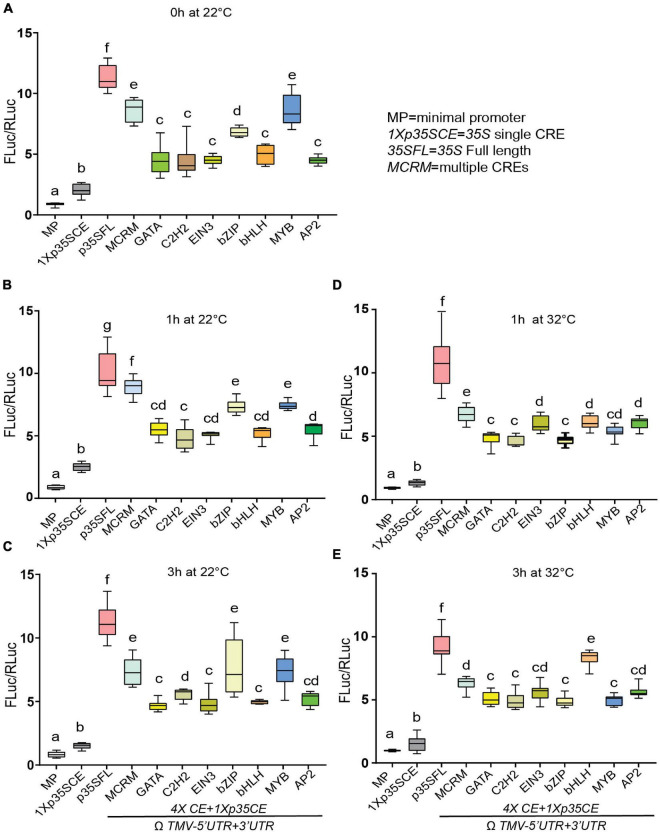
Effect of elevated temperature on the efficacies of synthetic promoters derived from CREs of core photorespiratory genes. **(A)** Relative luciferase activity driven by synthetic promoters under ambient condition at 0 h. **(B)** Relative luciferase activity driven by synthetic promoters under ambient condition at 1 h. **(C)** Relative luciferase activity driven by synthetic promoters under ambient condition at 3 h. **(D)** Relative luciferase activity driven by synthetic promoters in response to elevated temperature (32°C) for 1 h. **(E)** Relative luciferase activity driven by synthetic promoters in response to elevated temperature (32°C) for 3 h. Constructs (see Figure 5A) were agroinfiltrated and 2-day post infiltration one set of transformed plants were shifted to a growth chamber with elevated temperature (32°C) for 1 h, and 3 h. After 3 h, plants treated at high temperature are shifted back to ambient condition for 1.5 h (1.5hR). Control plants were kept at 22°C. The UTRs used are indicated by the line at the bottom. Data analyzed as described in Figure 1. Box-whiskers plots represent data from three independent experiments, each with three biological replicates. Error bar represents a mean ± standard deviation. Letters indicate statistical significance based on a two-way ANOVA with Tukey’s multiple component analysis (*p* < 0.05); samples sharing letters are not significantly different. Acronyms used are defined in the ‘Materials and Methods’ section.

Next, under ambient conditions (22°C), *MCRM* displayed heightened luciferase activity (∼8-fold) compared to synthetic promoters with single CRE (∼5-fold) except for *MYB-CRE* (Figures 6A–C). Among the different classes of single CREs, *MYB-CRE* displayed the strongest luciferase activity (∼8 fold) followed by *bZIP-CRE* (6.5-fold) under ambient conditions (22°C). No discernable change in luciferase activity was observed under ambient conditions over time in any of the promoter combinations, except for *GATA-*CRE (Figures 6B,C and Supplementary Figure S6). Next, we examined the effect of elevated temperature (32°C) on luciferase activity driven by these synthetic promoters. Interestingly, 1 h post elevated temperature stress, *MCRM* (∼15%), *MYB-*CRE (∼10%) and *bZIP-*CRE (∼6%) displayed reduced luciferase activity, compared to ambient conditions (Figure 6D). At 3 h post elevated temperature treatment, *bHLH*-CRE showed the highest luciferase activity (∼40%) (Figure 6E). However, none of the other *CREs* exhibit any significant change in luciferase activity, irrespective of the time-points. Contrary to *bHLH*-CRE, a modest reduction in luciferase activity was observed in *MCRM* (∼20%), *MYB*-CRE (∼30%) and *bZIP*-CRE (∼10%) in response to 3 h of elevated temperature stress.

**FIGURE 7:**
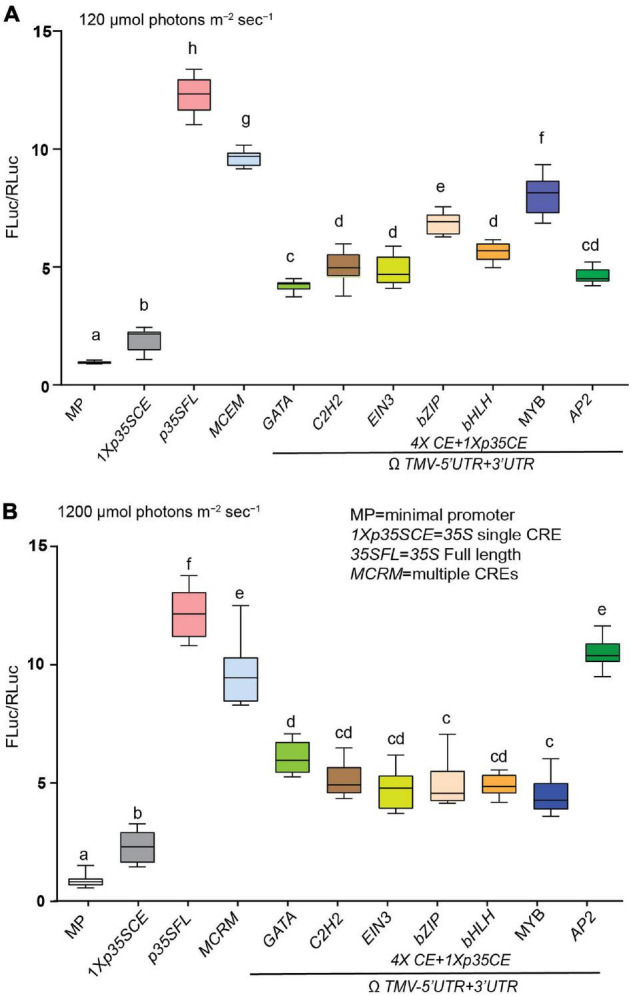
Effect of high light intensity on the efficacies of synthetic promoters derived from CREs of core photorespiratory genes. **(A)** Relative luciferase activity driven by synthetic promoters under ambient condition (150 μmol photons m^–2^ s^–1^). **(B)** Relative luciferase activity driven by synthetic promoters under ambient condition (1200 μmol photons m^–2^ s^–1^). Constructs (see Figure 5A) were agroinfiltrated and 2-day post infiltration one set of transformed plants were subjected to light intensity 1200 μmol photons m^–2^ s^–1^ and control transformed plants were maintained at 150 μmol photons m^–2^ s^–1^. Data analyzed as described in Figure 1. The UTRs used are indicated by the line at the bottom. Box-whiskers plots represent data from three independent experiments, each with three biological replicates. Error bar represents a mean ± standard deviation. Letters in the indicate statistical significance based on a one-way ANOVA with Tukey’s multiple component analysis (*p* < 0.05); samples sharing letters are not significantly different. Acronyms used are defined in the ‘Materials and Methods’ section.

Besides low CO_2_ and elevated temperature, photorespiration is modulated in response to high light intensity stress. This prompted us to test the response of high light intensity on the strength of these CREs driving luciferase activity. Consistent with previous data Figure 5, *MCRM* (∼10-fold) followed by *MYB-CRE* (∼8-fold) exhibited higher luciferase activity, under ambient conditions (Figure 7A). In response to high light intensity stress, *AP2*-CRE (∼30%) and *GATA*-CRE (∼5%) displayed higher luciferase activity, compared to ambient conditions (Figure 7B). Similar to elevated temperature, MCRM high light intensity stress also exhibited a modest reduction in luciferase activity (∼15%), compared to ambient conditions.

Except for modest reduction of luciferase activity driven by *p35SFL* (25%) in response to elevated temperature compared to 22°C, a heightened luciferase activity was observed irrespective of treatments and time points (Figures 5–7 and Supplementary Figure S6). Consistently, the lowest luciferase activity was displayed by *MP* followed *1Xp35SCE*, in all the conditions and time points tested and no discernable change in luciferase activity was observed under any stress conditions (Figures 5–7). Overall, the data presented in Figures 5–7 supports the hypothesis that most if not all CREs differentially influence luciferase activity under the three stress conditions, albeit to varying extents. These results imply that these motifs play a critical role for *PLGG1*, *BASS6*, and *PGLP* in mediating response to elevated temperature and high-intensity light.

## Discussion

It has been clearer that the possession of a well-equipped toolbox of promoters, UTRs, CREs and protein targeting signaling peptides is vital to achieving gene stacking for manipulating complex agronomic traits. For rapid high throughput analysis of GREs, a tobacco transient expression system was adopted to gauge the efficacies of GREs in a non-invasive reliable manner. Undoubtedly, generating transgenic lines, especially non-model plants, is quite a time-consuming and labor-intensive affair. Therefore, it is wise to examine the efficacies of the promoter-UTR combinations in a transient assay before embarking on generating stable transgenic crops. Here, we have provided a comprehensive comparison between a suite of constitutive promoters of various origins within a single assay (Figure 1). Most studies dealing with examining genetic elements for gene expression focus only on promoters. However, several reports have demonstrated that upstream and downstream untranslated regions play a critical role in influencing the expression of a transgene (Jeong et al., 2006; Bartlett et al., 2009; Karthikeyan et al., 2009; Matsui et al., 2012; Diamos and Mason, 2018; Srivastava et al., 2018; Yamasaki et al., 2018a). To this end, we have demonstrated that plant-derived UTRs outperformed viral UTRs under ambient conditions, consistent with previous findings (Figures 2, 3; Nagaya et al., 2010). However, plant-derived UTRs are more sensitive to environmental stress conditions compared to viral-UTRs (Supplementary Figures S3, S4). One of the key concepts of synthetic biology is the development of modular parts that can be used in different contexts, to this end, we have successfully generated new promoter-UTR combinations by combining plant UTRs with non-plant promoters (Figure 3). Since the final aim of crop engineering is to create improved lines, it is imperative that these transgenic lines be tested under field conditions. Field-grown plants frequently encounter elevated temperature and high irradiance assaults both of which can adversely affect photosynthetic efficiency and reduce yield significantly (Lu et al., 2017; Moore et al., 2021). Therefore, by testing the variation in luciferase activity driven by new promoter-UTR combinations under elevated temperature and high light intensity conditions we have presented several options for tailoring transcriptional activity for genetic circuits (Supplementary Figures S3, S4).

Constitutive promoters offer an opportunity for overexpression, but they have their limitations. For instance, constitutive promoters often remain associated with stress tolerance and defense responses. Usage of such promoters may lead to unintended phenotypes, including unexpected activation of defense pathways in the absence of pathogens, and in turn incurs fitness costs (Hernandez-Garcia and Finer, 2014; Ali and Kim, 2019). Furthermore, the lack of spatiotemporal regulations offered by constitutive promoters makes understanding of native promoters an obvious alternative (Ali and Kim, 2019; Cai et al., 2020). However, since native promoters tend to contain multiple functional CREs it is difficult to dissect the role of each of these CREs. Synthetic promoters containing native CREs provide an excellent solution as they lack the complexity of native promoters enabling us to fine-tune the expression pattern of a desired gene or genes in a predictable way. Synthetic promoters based on native CREs can allow us to investigate the roles of specific CREs and shed some light on their cognate transcriptional modulators under both ambient and stress conditions effectively. There have been previous reports on the identification and functional characterization of native CREs using specific key genes to gain insights into a complicated defense response or metabolic pathways (Kaplan et al., 2006; Kaur et al., 2017; Kakei et al., 2021). To the best of our knowledge, no study has been reported on synthetic promoters based on key photorespiratory genes from *Arabidopsis*. Photorespiration offers an attractive test case as it is intricately associated with the efficiency of photosynthesis, nitrogen metabolism, and stress responses (Voss et al., 2013; Bloom, 2015; Sunil et al., 2019).

In addition to considerable progress made in the functional characterization of PR genes, few studies have been dedicated to analyzing the regulation of photorespiratory genes at the promoter level (Vauclare et al., 1998; Laxa et al., 2016; Laxa, 2017). Since no common CRE was identified upon examining all characterized PR genes, we decided to focus on key genes (*PGLP*, *PLGG1* and *BASS6*) involved in the initial steps of the process (Laxa and Fromm, 2018). We demonstrated that in response to both elevated temperature and high light intensity stress conditions, *MYB*-CRE and *bZIP*-CRE displayed a reduction in luciferase activity most likely by recruiting transcriptional repressors (Figures 6, 7). On the contrary, *bHLH*-CRE and *AP2*-CRE represent predominantly positive influencers of luciferase activity in response to elevated temperature and high irradiance, respectively. It is important to note that in creating *MCRM*, we generated one construct without prioritizing motif location and, thereby cannot infer the positional effect of each element with respect to its neighboring motif (Cai et al., 2020). Since we have characterized the seven stress-responsive CREs shared by the PR genes using a transient reporter assay in tobacco, we relied on the endogenous transcription factors of tobacco as opposed to orthogonal transcription factors. A strong support for our *in silico* analysis was provided by the RNA-seq analysis performed using known PR mutants in response to a short-term reduction of the external CO_2_ concentration (Eisenhut et al., 2017). Consistent with our findings, Eisenhut et al. (2017) reported that AP2, bHLH, and C2H2 as differentially upregulated transcription factors while *bZIP* as one of the predominantly downregulated transcription factors. The bZIP and MYB transcription factors have been implicated to be downregulated in response to heat stress (Yang et al., 2019; Zhang et al., 2019; Liu et al., 2021).

While many of the photorespiratory genes were shown to be are transcriptionally responsive to light, nutrients, metabolites and pathogens, the underlying mechanism of integrating these signals on the promoters of the key genes are highly complex and poorly understood (Laxa and Fromm, 2018). We expected an induction in *NbPGLP* upon high temperature stress, as it is widely accepted that rate of photorespiration increases at elevated temperature. Intriguingly, we observed downregulation of *NbPGLP* in response to high temperature and high light intensity stresses (Figures 4B,C). Contrary to our findings, Timm et al., 2019 reported a modest increase in *PGLP* expression levels in Arabidopsis after 1-and 3-day of high temperature stress at 30°C. Such difference in the *PGLP* expression profile may be attributed to the difference in short-term versus long-term response to elevated temperature. Furthermore, after 7-day of high temperature treatment reduction in PGLP expression levels was observed. Another independent study demonstrated the induction of *PGLP* in response to elevated temperature in Poplar trees (Song et al., 2014). Such disparity in differential thermal response may be attributed to widely diverse species and the adoption of promoter elements from different species could lead to differential expression needed in synthetic biology designs. Low [CO_2_] also triggers higher photorespiratory rates but at 200 ppm of CO_2_, we did not detect any discernable difference in the transcript levels of any of the three genes tested (Figure 4A). One plausible explanation is that 200 ppm may not be a low enough [CO_2_] level to trigger a transcriptional change in any of these genes.

Collectively this study presents a versatile, toolbox for multigene transformation that will not benefit designing alternative photorespiratory bypasses but also can be adopted for various synthetic biology approaches in plant biotechnology.

## Data Availability Statement

The raw data supporting the conclusions of this article will be made available by the authors, without undue reservation.

## Author Contributions

DB and PS designed the experiments, analyzed the data, and wrote the manuscript. DB performed the experiments. Both authors contributed to the article and approved the submitted version.

## Conflict of Interest

The authors declare that the research was conducted in the absence of any commercial or financial relationships that could be construed as a potential conflict of interest.

## Publisher’s Note

All claims expressed in this article are solely those of the authors and do not necessarily represent those of their affiliated organizations, or those of the publisher, the editors and the reviewers. Any product that may be evaluated in this article, or claim that may be made by its manufacturer, is not guaranteed or endorsed by the publisher.

## Footnotes

1
http://www.athamap.de/search_gene.php

2
http://PlantPAN3.itps.ncku.edu.tw

## Supplementary Material

Supplementary Table S1 Details of L0, L1 and L2 plasmids used in this study.

## References

[B1] Agnolucci P. Rapti C. Alexander P. De Lipsis V. Holland R. A. Eigenbrod F. (2020). Impacts of rising temperatures and farm management practices on global yields of 18 crops. *Nat. Food* 1 562–571. 10.1038/s43016-020-00148-x 37128016

[B2] Ali S. Kim W.-C. (2019). A fruitful decade using synthetic promoters in the improvement of transgenic plants. *Front. Plant Sci.* 10:1433. 10.3389/fpls.2019.01433 31737027 PMC6838210

[B3] Amack S. C. Antunes M. S. (2020). CaMV35S promoter – A plant biology and biotechnology workhorse in the era of synthetic biology. *Curr. Plant Biol.* 24:100179. 10.1016/j.cpb.2020.100179

[B4] An Y. Q. McDowell J. M. Huang S. McKinney E. C. Chambliss S. Meagher R. B. (1996). Strong, constitutive expression of the Arabidopsis ACT2/ACT8 actin subclass in vegetative tissues. *Plant J.* 10 107–121. 10.1046/j.1365-313x.1996.10010107.x 8758981

[B5] Anderson C. M. Mattoon E. M. Zhang N. Becker E. McHargue W. Yang J. (2021). High light and temperature reduce photosynthetic efficiency through different mechanisms in the C4 model Setaria viridis. *Commun. Biol.* 4:1092. 10.1038/s42003-021-02576-2 34531541 PMC8446033

[B6] Balfagón D. Sengupta S. Gómez-Cadenas A. Fritschi F. B. Azad R. K. Mittler R. (2019). Jasmonic acid is required for plant acclimation to a combination of high light and heat stress. *Plant Physiol.* 181 1668–1682. 10.1104/pp.19.00956 31594842 PMC6878009

[B7] Bartlett J. G. Snape J. W. Harwood W. A. (2009). Intron-mediated enhancement as a method for increasing transgene expression levels in barly. *Plant Biotechnol. J*. 7, 856–866. 10.1111/j.1467-7652.2009.00448.x 19781005

[B8] Batista-Silva W. da Fonseca-Pereira P. Martins A. O. Zsögön A. Nunes-Nesi A. Araújo W. L. (2020). Engineering improved photosynthesis in the era of synthetic biology. *Plant Commun.* 1:100032. 10.1016/j.xplc.2020.100032 33367233 PMC7747996

[B9] Bauwe H. Hagemann M. Kern R. Timm S. (2012). Photorespiration has a dual origin and manifold links to central metabolism. *Curr. Opin. Plant Biol.* 15 269–275. 10.1016/j.pbi.2012.01.008 22284850

[B10] Behera S. Wang N. Zhang C. Schmitz-Thom I. Strohkamp S. Schültke S. (2015). Analyses of Ca2+ dynamics using a ubiquitin-10 promoter-driven Yellow Cameleon 3.6 indicator reveal reliable transgene expression and differences in cytoplasmic Ca2+ responses in Arabidopsis and rice (Oryza sativa) roots. *New Phytol.* 206 751–760. 10.1111/nph.13250 25641067

[B11] Beringer J. Chen W. Garton R. Sardesai N. Wang P.-H. Zhou N. (2017). Comparison of the impact of viral and plant-derived promoters regulating selectable marker gene on maize transformation and transgene expression. *Plant Cell Rep.* 36 519–528. 10.1007/s00299-017-2099-y 28160062 PMC5360835

[B12] Bernardes W. S. Menossi M. (2020). Plant 3′ Regulatory Regions From mRNA-Encoding Genes and Their Uses to Modulate Expression. *Front. Plant Sci.* 11:1252. 10.3389/fpls.2020.01252 32922424 PMC7457121

[B13] Betts S. D. Basu S. Bolar J. Booth R. Chang S. Cigan A. M. (2019). Uniform expression and relatively small position effects characterize sister transformants in maize and soybean. *Front. Plant Sci.* 10:1209. 10.3389/fpls.2019.01209 31708936 PMC6821721

[B14] Bloom A. J. (2015). Photorespiration and nitrate assimilation: a major intersection between plant carbon and nitrogen. *Photosynt. Res.* 123 117–128. 10.1007/s11120-014-0056-y 25366830

[B15] Boyko A. Molinier J. Chatter W. Laroche A. Kovalchuk I. (2010). Acute but not chronic exposure to abiotic stress results in transient reduction of expression levels of the transgene driven by the 35S promoter. *New Biotechnol.* 27 70–77. 10.1016/j.nbt.2009.09.007 19800040

[B16] Cai Y.-M. Kallam K. Tidd H. Gendarini G. Salzman A. Patron N. J. (2020). Rational design of minimal synthetic promoters for plants. *Nucleic Acids Res.* 48 11845–11856. 10.1093/nar/gkaa682 32856047 PMC7708054

[B17] Chow C.-N. Lee T.-Y. Hung Y.-C. Li G.-Z. Tseng K.-C. Liu Y.-H. (2019). PlantPAN3.0: a new and updated resource for reconstructing transcriptional regulatory networks from ChIP-seq experiments in plants. *Nucleic Acids Res.* 47 D1155–D1163. 10.1093/nar/gky1081 30395277 PMC6323957

[B18] Chow C.-N. Zheng H.-Q. Wu N.-Y. Chien C.-H. Huang H.-D. Lee T.-Y. (2016). PlantPAN 2.0: an update of plant promoter analysis navigator for reconstructing transcriptional regulatory networks in plants. *Nucleic Acids Res.* 44 D1154–D1160. 10.1093/nar/gkv1035 26476450 PMC4702776

[B19] Christensen A. H. Sharrock R. A. Quail P. H. (1992). Maize polyubiquitin genes: structure, thermal perturbation of expression and transcript splicing, and promoter activity following transfer to protoplasts by electroporation. *Plant Mol. Biol.* 18 675–689. 10.1007/BF00020010 1313711

[B20] Daniell H. Dhingra A. (2002). Multigene engineering: dawn of an exciting new era in biotechnology. *Curr. Opin. Biotechnol.* 13 136–141. 10.1016/s0958-1669(02)00297-5 11950565 PMC3481857

[B21] De Bolle M. F. C. Butaye K. M. J. Coucke W. J. W. Goderis I. J. W. M. Wouters P. F. J. van Boxel N. (2003). Analysis of the influence of promoter elements and a matrix attachment region on the inter-individual variation of transgene expression in populations of Arabidopsis thaliana. *Plant Sci.* 165 169–179.

[B22] de Felippes F. McHale M. Doran R. L. Roden S. Eamens A. L. Finnegan E. J. (2020). The key role of terminators on the expression and post-transcriptional gene silencing of transgenes. *Plant J.* 104 96–112. 10.1111/tpj.14907 32603508

[B23] Diamos A. G. Mason H. S. (2018). Chimeric 3′ flanking regions strongly enhance gene expression in plants. *Plant Biotechnol. J.* 16 1971–1982. 10.1111/pbi.12931 29637682 PMC6230951

[B24] Eisenhut M. Bräutigam A. Timm S. Florian A. Tohge T. Fernie A. R. (2017). Photorespiration is crucial for dynamic response of photosynthetic metabolism and stomatal movement to altered CO(2) availability. *Mol. Plant* 10 47–61. 10.1016/j.molp.2016.09.011 27702693

[B25] Engler C. Youles M. Gruetzner R. Ehnert T.-M. Werner S. Jones J. D. G. (2014). A golden gate modular cloning toolbox for plants. *ACS Synthetic Biol.* 3 839–843. 10.1021/sb4001504 24933124

[B26] Eseverri Á López-Torrejón G. Jiang X. Burén S. Rubio L. M. Caro E. (2020). Use of synthetic biology tools to optimize the production of active nitrogenase Fe protein in chloroplasts of tobacco leaf cells. *Plant Biotechnol. J.* 18 1882–1896. 10.1111/pbi.13347 31985876 PMC7415783

[B27] Estrada-Melo A. C. Chao, Reid M. S. Jiang C.-Z. (2015). Overexpression of an ABA biosynthesis gene using a stress-inducible promoter enhances drought resistance in petunia. *Horticult. Res.* 2:15013. 10.1038/hortres.2015.13 26504568 PMC4595983

[B28] Fan Q. Treder K. Miller W. A. (2012). Untranslated regions of diverse plant viral RNAs vary greatly in translation enhancement efficiency. *BMC Biotechnol.* 12:22. 10.1186/1472-6750-12-22 22559081 PMC3416697

[B29] Feike D. Korolev A. V. Soumpourou E. Murakami E. Reid D. Breakspear A. (2019). Characterizing standard genetic parts and establishing common principles for engineering legume and cereal roots. *Plant Biotechnol. J.* 17 2234–2245. 10.1111/pbi.13135 31022324 PMC6835126

[B30] Gallie D. R. Walbot V. (1992). Identification of the motifs within the tobacco mosaic virus 5′-leader responsible for enhancing translation. *Nucleic Acids Res.* 20 4631–4638. 10.1093/nar/20.17.4631 1408765 PMC334194

[B31] Grefen C. Donald N. Hashimoto K. Kudla J. Schumacher K. Blatt M. R. (2010). A ubiquitin-10 promoter-based vector set for fluorescent protein tagging facilitates temporal stability and native protein distribution in transient and stable expression studies. *Plant J.* 64 355–365. 10.1111/j.1365-313X.2010.04322.x 20735773

[B32] Halpin C. (2005). Gene stacking in transgenic plants – the challenge for 21st century plant biotechnology. *Plant Biotechnol. J.* 3 141–155. 10.1111/j.1467-7652.2004.00113.x 17173615

[B33] Hatfield J. L. Prueger J. H. (2015). Temperature extremes: Effect on plant growth and development. *Weather Clim. Extremes* 10 4–10. 10.1016/j.wace.2015.08.001

[B34] Hehl R. Norval L. Romanov A. Bülow L. (2016). Boosting AthaMap database content with data from protein binding microarrays. *Plant Cell Physiol.* 57:e4. 10.1093/pcp/pcv156 26542109

[B35] Hernandez-Garcia C. M. Finer J. J. (2014). Identification and validation of promoters and cis-acting regulatory elements. *Plant Sci.* 21 109–119. 10.1016/j.plantsci.2013.12.007 24467902

[B36] Huang W. Hu H. Zhang S.-B. (2015). Photorespiration plays an important role in the regulation of photosynthetic electron flow under fluctuating light in tobacco plants grown under full sunlight. *Front. Plant Sci.* 6:621. 10.3389/fpls.2015.00621 26322062 PMC4531324

[B37] Jeong Y.-M. Mun J.-H. Lee I. Woo J. C. Hong C. B. Kim S. G. (2006). Distinct roles of the first introns on the expression of *Arabidopsis* profilin gene family members. *Plant Physiol*. 140, 196–209. 10.1104/pp.105.071316 16361517 PMC1326044

[B38] Jiang P. Zhang K. Ding Z. He Q. Li W. Zhu S. (2018). Characterization of a strong and constitutive promoter from the Arabidopsis serine carboxypeptidase-like gene AtSCPL30 as a potential tool for crop transgenic breeding. *BMC Biotechnol.* 18:59. 10.1186/s12896-018-0470-x 30241468 PMC6151023

[B39] Jores T. Tonnies J. Wrightsman T. Buckler E. S. Cuperus J. T. Fields S. (2021). Synthetic promoter designs enabled by a comprehensive analysis of plant core promoters. *Nat. Plants* 7 842–855. 10.1038/s41477-021-00932-y 34083762 PMC10246763

[B40] Kakei Y. Masuda H. Nishizawa N. K. Hattori H. Aung M. S. (2021). Elucidation of novel cis-regulatory elements and promoter structures involved in iron excess response mechanisms in rice using a bioinformatics approach. *Front. Plant Sci.* 12:766. 10.3389/fpls.2021.660303 34149757 PMC8207140

[B41] Kaplan B. Davydov O. Knight H. Galon Y. Knight M. R. Fluhr R. (2006). Rapid transcriptome changes induced by cytosolic Ca2+ transients reveal ABRE-related sequences as Ca2+-responsive cis elements in Arabidopsis. *Plant Cell* 18 2733–2748. 10.1105/tpc.106.042713 16980540 PMC1626612

[B42] Karthikeyan A. S. Ballachanda D. N. Raghothama K. G. (2009). Promoter deletion analysis elucidates the role of cis elements and 5′UTR intron in spatiotemporal regulation of *AtPht1;4* expression in *Arabidopsis*. *Physiol. Plant* 136, 10–18. 10.1111/j.1399-3054.2009.01207.x 19508364

[B43] Kaur A. Pati P. K. Pati A. M. Nagpal A. K. (2017). In-silico analysis of cis-acting regulatory elements of pathogenesis-related proteins of Arabidopsis thaliana and Oryza sativa. *PLoS One* 12:e0184523. 10.1371/journal.pone.0184523 28910327 PMC5598985

[B44] Kebeish R. Niessen M. Thiruveedhi K. Bari R. Hirsch H.-J. Rosenkranz R. (2007). Chloroplastic photorespiratory bypass increases photosynthesis and biomass production in Arabidopsis thaliana. *Nat. Biotechnol.* 25 593–599. 10.1038/nbt1299 17435746

[B45] Koncz C. De Greve H. André D. Deboeck F. Van Montagu M. Schell J. (1983). The opine synthase genes carried by Ti plasmids contain all signals necessary for expression in plants. *EMBO J.* 2 1597–1603. 10.1002/j.1460-2075.1983.tb01630.x 11892818 PMC555329

[B46] Lassen J. Madsen K. Sandøe P. (2002). Ethics and genetic engineering - Lessons to be learned from GM foods. *Bioproc. Biosyst. Engine.* 24 263–271. 10.1007/s004490100262

[B47] Laxa M. (2017). Regulatory cis-elements are located in accessible promoter regions of the CAT2 promoter and affect activating histone modifications in Arabidopsis thaliana. *Plant Mol. Biol.* 93 49–60. 10.1007/s11103-016-0546-8 27734290

[B48] Laxa M. Fromm S. (2018). Co-expression and regulation of photorespiratory genes in Arabidopsis thaliana: A bioinformatic approach. *Curr. Plant Biol.* 14 2–18. 10.1016/j.cpb.2018.09.001

[B49] Laxa M. Müller K. Lange N. Doering L. Pruscha J. T. Peterhänsel C. (2016). The 5′UTR Intron of Arabidopsis GGT1 Aminotransferase Enhances Promoter Activity by Recruiting RNA Polymerase II. *Plant Physiol.* 172 313–327. 10.1104/pp.16.00881 27418588 PMC5074633

[B50] Liu B. Kong L. Zhang Y. Liao Y. (2021). Gene and metabolite integration analysis through transcriptome and metabolome brings new insight into heat stress tolerance in potato (Solanum tuberosum l.). *Plants* 10 1–17. 10.3390/plants10010103 33419030 PMC7825342

[B51] Liu D. Shi L. Han C. Yu J. Li D. Zhang Y. (2012). Validation of reference genes for gene expression studies in virus-infected Nicotiana benthamiana using quantitative real-time PCR. *PLoS One* 7:e46451. 10.1371/journal.pone.0046451 23029521 PMC3460881

[B52] Livak K. J. Schmittgen T. D. (2001). Analysis of relative gene expression data using real-time quantitative PCR and the 2−ΔΔCT method. *Methods* 25 402–408. 10.1006/meth.2001.1262 11846609

[B53] Lozada D. N. Mason R. E. Babar M. A. Carver B. F. Guedira G.-B. Merrill K. (2017). Association mapping reveals loci associated with multiple traits that affect grain yield and adaptation in soft winter wheat. *Euphytica* 213:222.

[B54] Lu T. Meng Z. Zhang G. Qi M. Sun Z. Liu Y. (2017). Sub-high temperature and high light intensity induced irreversible inhibition on photosynthesis system of tomato plant (Solanum lycopersicum L.). *Front. Plant Sci.* 8:365. 10.3389/fpls.2017.00365 28360922 PMC5352666

[B55] Ma Y. Chhapekar S. S. Rameneni J. J. Kim S. Gan T. H. Choi S. R. (2021). Identification of qtls and candidate genes related to flower traits and bolting time in radish (Raphanus sativus l.). *Agronomy* 11 1–16.

[B56] Manickavasagam M. Ganapathi A. Anbazhagan V. R. Sudhakar B. Selvaraj N. Vasudevan A. (2004). Agrobacterium-mediated genetic transformation and development of herbicide-resistant sugarcane (Saccharum species hybrids) using axillary buds. *Plant Cell Rep.* 23 134–143. 10.1007/s00299-004-0794-y 15133712

[B57] Martìnez-Hernaàndez A. Loàpez-Ochoa L. Argüello-Astorga G. Herrera-Estrella L. (2002). Functional properties and regulatory complexity of a minimalRBCS light-responsive unit activated by phytochrome, cryptochrome, and plastid signals. *Plant Physiol.* 128 1223–1233. 10.1104/pp.010678 11950971 PMC154250

[B58] Matsui T. Matsuura H. Sawada K. Takita E. Kinjo S. Takenami S. (2012). High level expression of transgenes by use of 5′-untranslated region of the Arabidopsis thaliana arabinogalactan-protein 21 gene in dicotyledons. *Plant Biotechnol.* 29 319–322. 10.5511/plantbiotechnology.12.0322a

[B59] Matzke M. A. Matzke A. J. M. (1995). How and why do plants inactivate homologous (trans)genes? *Plant Physiol.* 107 679–685. 10.1104/pp.107.3.679 12228391 PMC157182

[B60] McElroy D. Zhang W. Cao J. Wu R. (1990). Isolation of an efficient actin promoter for use in rice transformation. *Plant Cell* 2 163–171. 10.1105/tpc.2.2.163 2136633 PMC159873

[B61] Melo B. P. de, Moura S. M. de, Morgante C. V. Pinheiro D. H. (2021). Regulated promoters applied to plant engineering: an insight over promising soybean promoters under biotic stress and their cis-elements. *Biotechnol. Res. Innovat.* 5:e2021005. 10.4322/biori.202105

[B62] Moellenbeck D. J. Peters M. L. Bing J. W. Rouse J. R. Higgins L. S. Sims L. (2001). Insecticidal proteins from Bacillus thuringiensis protect corn from corn rootworms. *Nat. Biotechnol.* 19 668–672. 10.1038/90282 11433280

[B63] Moore C. E. Meacham-Hensold K. Lemonnier P. Slattery R. A. Benjamin C. Bernacchi C. J. (2021). The effect of increasing temperature on crop photosynthesis: From enzymes to ecosystems. *J. Exp. Bot.* 72 2822–2844. 10.1093/jxb/erab090 33619527 PMC8023210

[B64] Nagaya S. Kawamura K. Shinmyo A. Kato K. (2010). The HSP Terminator of Arabidopsis thaliana Increases Gene Expression in Plant Cells. *Plant Cell Physiol.* 51 328–332. 10.1093/pcp/pcp188 20040586

[B65] Obertello M. Santi C. Sy M.-O. Laplaze L. Auguy F. Bogusz D. (2005). Comparison of four constitutive promoters for the expression of transgenes in the tropical nitrogen-fixing tree Allocasuarina verticillata. *Plant Cell Rep.* 24 540–548. 10.1007/s00299-005-0963-7 15940528

[B66] Odell J. T. Nagy F. Chua N.-H. (1985). Identification of DNA sequences required for activity of the cauliflower mosaic virus 35S promoter. *Nature* 313 810–812. 10.1038/313810a0 3974711

[B67] Oliveira P. H. Prather K. J. Prazeres D. M. F. Monteiro G. A. (2010). Analysis of DNA repeats in bacterial plasmids reveals the potential for recurrent instability events. *Appl. Microbiol. Biotechnol*. 87, 2157–2167. 10.1007/s00253-010-2671-7 20496146

[B68] Ort D. R. Merchant S. S. Alric J. Barkan A. Blankenship R. E. Bock R. (2015). Redesigning photosynthesis to sustainably meet global food and bioenergy demand. *Proc. Natl. Acad. Sci.* 112 8529L–8536. 10.1073/pnas.1424031112 26124102 PMC4507207

[B69] Park S.-H. Yi N. Kim Y. S. Jeong M.-H. Bang S.-W. Choi Y. (2010). Analysis of five novel putative constitutive gene promoters in transgenic rice plants. *J. Exp. Bot.* 61 2459–2467. 10.1093/jxb/erq076 20363869 PMC2877896

[B70] Patron N. J. Orzaez D. Marillonnet S. Warzecha H. Matthewman C. Youles M. (2015). Standards for plant synthetic biology: a common syntax for exchange of DNA parts. *New Phytol.* 208 13–19. 10.1111/nph.13532 26171760

[B71] Peramuna A. Bae H. Rasmussen E. K. Dueholm B. Waibel T. Critchley J. H. (2018). Evaluation of synthetic promoters in Physcomitrella patens. *Biochem. Biophys. Res. Commun.* 500 418–422. 10.1016/j.bbrc.2018.04.092 29660341

[B72] Peremarti A. Twyman R. M. Gómez-Galera S. Naqvi S. Farré G. Sabalza M. (2010). Promoter diversity in multigene transformation. *Plant Mol. Biol.* 73 363–378. 10.1007/s11103-010-9628-1 20354894

[B73] Pick T. R. Bräutigam A. Schulz M. A. Obata T. Fernie A. R. Weber A. P. M. (2013). PLGG1, a plastidic glycolate glycerate transporter, is required for photorespiration and defines a unique class of metabolite transporters. *Proc. Natl. Acad. Sci.* 110 3185L–3190. 10.1073/pnas.1215142110 23382251 PMC3581909

[B74] Rojas C. M. Senthil-Kumar M. Wang K. Ryu C.-M. Kaundal A. Mysore K. S. (2012). Glycolate oxidase modulates reactive oxygen species–mediated signal transduction during nonhost resistance in Nicotiana benthamiana and Arabidopsis. *Plant Cell* 24 336–352. 10.1105/tpc.111.093245 22286136 PMC3289552

[B75] Saji S. Bathula S. Kubo A. Tamaoki M. Aono M. Sano T. (2017). Ozone-sensitive Arabidopsis mutants with deficiencies in photorespiratory enzymes. *Plant Cell Physiol.* 58 914–924. 10.1093/pcp/pcx027 28339978

[B76] Sanders P. R. Winter J. A. Barnason A. R. Rogers S. G. Fraley R. T. (1987). Comparison of cauliflower mosaic virus 35S and nopaline synthase promoters in transgenic plants. *Nucleic Acids Res.* 15 1543–1558. 10.1093/nar/15.4.1543 3029718 PMC340566

[B77] Satoh J. Kato K. Shinmyo A. (2004). The 5′-untranslated region of the tobacco alcohol dehydrogenase gene functions as an effective translational enhancer in plant. *J. Biosci. Bioengine.* 98 1–8. 10.1016/S1389-1723(04)70234-0 16233658

[B78] Schwarte S. Bauwe H. (2007). Identification of the photorespiratory 2-phosphoglycolate phosphatase, PGLP1, in Arabidopsis. *Plant Physiol.* 144 1580–1586. 10.1104/pp.107.099192 17478634 PMC1914141

[B79] Sharkey T. D. (1988). Estimating the rate of photorespiration in leaves. *Physiol. Plant.* 73 147–152. 10.1111/j.1438-8677.2012.00694.x 23186383

[B80] Shen B.-R. Wang L.-M. Lin X.-L. Yao Z. Xu H.-W. Zhu C.-H. (2019). Engineering a new chloroplastic photorespiratory bypass to increase photosynthetic efficiency and productivity in rice. *Mol. Plant* 12 199–214. 10.1016/j.molp.2018.11.013 30639120

[B81] Slattery R. Ort D. (2019). Carbon assimilation in crops at high temperatures. *Plant Cell Environ.* 42:13572. 10.1111/pce.13572 31046135

[B82] Solberg N. Krauss S. (2013). Luciferase assay to study the activity of a cloned promoter DNA fragment. *Methods Mol. Biol.* 977 65–78. 10.1007/978-1-62703-284-1_6 23436354

[B83] Song Y. Chen Q. Ci D. Shao X. Zhang D. (2014). Effects of high temperature on photosynthesis and related gene expression in poplar. *BMC Plant Biol.* 14:111. 10.1186/1471-2229-14-111 24774695 PMC4036403

[B84] South P. F. Cavanagh A. P. Liu H. W. Ort D. R. (2019). Synthetic glycolate metabolism pathways stimulate crop growth and productivity in the field. *Science* 363:eaat9077. 10.1126/science.aat9077 PMC7745124 30606819

[B85] South P. F. Cavanagh A. P. Lopez-Calcagno P. E. Raines C. A. Ort D. R. (2018). Optimizing photorespiration for improved crop productivity. *J. Integrat. Plant Biol.* 60 1217–1230. 10.1111/jipb.12709 30126060

[B86] South P. F. Walker B. J. Cavanagh A. P. Rolland V. Badger M. Ort D. R. (2017). Bile acid sodium symporter BASS6 can transport glycolate and is involved in photorespiratory metabolism in Arabidopsis thaliana. *Plant Cell* 29 808–823. 10.1105/tpc.16.00775 28351992 PMC5435425

[B87] Srivastava A. K. Lu Y. Zinta G. Lang Z. Zhu J.-K. (2018). UTR-dependent control of gene expression in plants. *Trends Plant Sci.* 23 248–259. 10.1016/j.tplants.2017.11.003 29223924 PMC5828884

[B88] Steffens N. O. Galuschka C. Schindler M. Bülow L. Hehl R. (2005). AthaMap web tools for database-assisted identification of combinatorial cis-regulatory elements and the display of highly conserved transcription factor binding sites in Arabidopsis thaliana. *Nucleic Acids Res.* 33 W397–W402. 10.1093/nar/gki395 15980498 PMC1160156

[B89] Sunil B. Saini D. Bapatla R. B. Aswani V. Raghavendra A. S. (2019). Photorespiration is complemented by cyclic electron flow and the alternative oxidase pathway to optimize photosynthesis and protect against abiotic stress. *Photosynth. Res.* 139 67–79. 10.1007/s11120-018-0577-x 30187303

[B90] Timm S. Woitschach F. Heise C. Hagemann M. Bauwe H. (2019). Faster removal of 2-phosphoglycolate through photorespiration improves abiotic stress tolerance of Arabidopsis. *Plants* 8:563. 10.3390/plants8120563 31810232 PMC6963629

[B91] Tong Z. Hong B. Yang Y. Li Q. Ma N. Ma C. (2009). Overexpression of two chrysanthemum DgDREB1 group genes causing delayed flowering or dwarfism in Arabidopsis. *Plant Mol. Biol.* 71 115–129. 10.1007/s11103-009-9513-y 19544047

[B92] Vauclare P. Macherel D. Douce R. Bourguignon J. (1998). The gene encoding T protein of the glycine decarboxylase complex involved in the mitochondrial step of the photorespiratory pathway in plants exhibits features of light-induced genes. *Plant Mol. Biol.* 37 309–318. 10.1023/a:1005954200042 9617803

[B93] Voss I. Sunil B. Scheibe R. Raghavendra A. S. (2013). Emerging concept for the role of photorespiration as an important part of abiotic stress response. *Plant Biol.* 15 713–722. 10.1111/j.1438-8677.2012.00710.x 23452019

[B94] Wang X. Xu L. Liu X. Xin L. Wu S. Chen X. (2021). Development of potent promoters that drive the efficient expression of genes in apple protoplasts. *Horticult. Res.* 8:211. 10.1038/s41438-021-00646-4 34593780 PMC8484340

[B95] Wang Y. Wang C. Rajaofera M. J. N. Zhu L. Liu W. Zheng F. (2020). WY7 is a newly identified promoter from the rubber powdery mildew pathogen that regulates exogenous gene expression in both monocots and dicots. *PLoS One* 15:e0233911. 10.1371/journal.pone.0233911 32479550 PMC7263610

[B96] Weber E. Engler C. Gruetzner R. Werner S. Marillonnet S. (2011). A modular cloning system for standardized assembly of multigene constructs. *PLoS One* 6:e16765. 10.1371/journal.pone.0016765 21364738 PMC3041749

[B97] Wever W. McCallum E. J. Chakravorty D. Cazzonelli C. I. Botella J. R. (2010). The 5′ untranslated region of the VR-ACS1 mRNA acts as a strong translational enhancer in plants. *Transgenic Res.* 19 667–674. 10.1007/s11248-009-9332-6 19816782

[B98] Winter D. Vinegar B. Nahal H. Ammar R. Wilson G. V. Provart N. J. (2007). An “electronic fluorescent pictograph” browser for exploring and analyzing large-scale biological data sets. *PLoS One* 2:e718. 10.1371/journal.pone.0000718 17684564 PMC1934936

[B99] Wu Y. Wang Y. Li J. Li W. Zhang L. Li Y. (2014). Development of a general method for detection and quantification of the P35S promoter based on assessment of existing methods. *Sci. Rep.* 4:7358. 10.1038/srep07358 25483893 PMC4258656

[B100] Yamamoto T. Hoshikawa K. Ezura K. Okazawa R. Fujita S. Takaoka M. (2018). Improvement of the transient expression system for production of recombinant proteins in plants. *Sci. Rep.* 8:4755. 10.1038/s41598-018-23024-y 29555968 PMC5859073

[B101] Yamasaki S. Sanada Y. Imase R. Matsuura H. Ueno D. Demura T. (2018a). Arabidopsis thaliana cold-regulated 47 gene 5′-untranslated region enables stable high-level expression of transgenes. *J. Biosci. Bioengine.* 125 124–130. 10.1016/j.jbiosc.2017.08.007 28918993

[B102] Yamasaki S. Suzuki A. Yamano Y. Kawabe H. Ueno D. Demura T. (2018b). Identification of 5′-untranslated regions that function as effective translational enhancers in monocotyledonous plant cells using a novel method of genome-wide analysis. *Plant Biotechnol.* 35 365–373. 10.5511/plantbiotechnology.18.0903a 31892824 PMC6905215

[B103] Yang Q. Q. Feng K. Xu Z. S. Duan A. Q. Liu J. X. Xiong A. S. (2019). Genome-wide identification of bZIP transcription factors and their responses to abiotic stress in celery. *Biotechnol. Biotechnol. Equip.* 33 707–718. 10.1080/13102818.2019.1611386

[B104] Yu X. Klejnot J. Zhao X. Shalitin D. Maymon M. Yang H. (2007). Arabidopsis cryptochrome 2 completes its posttranslational life cycle in the nucleus. *Plant Cell* 19 3146–3156. 10.1105/tpc.107.053017 17965271 PMC2174722

[B105] Zhang M. Liu Y.-H. Xu W. Smith C. W. Murray S. C. Zhang H.-B. (2020). Analysis of the genes controlling three quantitative traits in three diverse plant species reveals the molecular basis of quantitative traits. *Sci. Rep.* 10:10074. 10.1038/s41598-020-66271-8 32572040 PMC7308372

[B106] Zhang S. Zhang A. Wu X. Zhu Z. Yang Z. Zhu Y. (2019). Transcriptome analysis revealed expression of genes related to anthocyanin biosynthesis in eggplant (Solanum melongena L.) under high-temperature stress. *BMC Plant Biol.* 19:387. 10.1186/s12870-019-1960-2 31492114 PMC6729041

[B107] Zhao C. Liu B. Piao S. Wang X. Lobell D. B. Huang Y. (2017). Temperature increase reduces global yields of major crops in four independent estimates. *Proc. Natl. Acad. Sci.* 114 9326L–9331. 10.1073/pnas.1701762114 28811375 PMC5584412

