# Supplementary material for: Design and Analysis of Native Photorespiration Gene Motifs of Promoter Untranslated Region Combinations Under Short Term Abiotic Stress Conditions
Source: Front Plant Sci. 2022 Feb 16;13:828729. doi: 10.3389/fpls.2022.828729 (PMC8888687; doi:10.3389/fpls.2022.828729)
Supplement: Supplementary file 2 [file Table_2.DOCX]

**Supplemental Table S2.** Sequences of oligonucleotides used in this study.

| Name | Primer 5’-3’ |
| --- | --- |
| *NbEF1αF* | AGCTTTACCTCCCAAGTCATCATGATG |
| *NbEF1αF* | CAAAACGTCCAATGGTGGGTACTCA |
| *NbPGLPF* | TGGACCGGAAGATGGTGACAAGAAAA |
| *NbPGLPR* | GAACCACCACCTGCCCATTCCTGA |
| *NbPLGG1F* | CATCTCTCACGGCAGCTGTTGTTGTA |
| *NbPLGG1R* | TGTCTTACTGCTGGAATGGAGCACAC |
| *NbBASS6F* | GCTTGTGATTGCATTTCATTTGTCAGCGT |
| *NbBASS6R* | CCACATCATTACTAGAGAGAAGCCCAT |
| *NbL23F* | AAGGATGCCGTGAAGATGT |
| *NbL23R* | AAATGAAAAACAGAAGGCACTTCCGT |
