# Supplementary figures and images for: Design and Analysis of Native Photorespiration Gene Motifs of Promoter Untranslated Region Combinations Under Short Term Abiotic Stress Conditions
Source: Front Plant Sci. 2022 Feb 16;13:828729. doi: 10.3389/fpls.2022.828729 (PMC8888687; doi:10.3389/fpls.2022.828729)

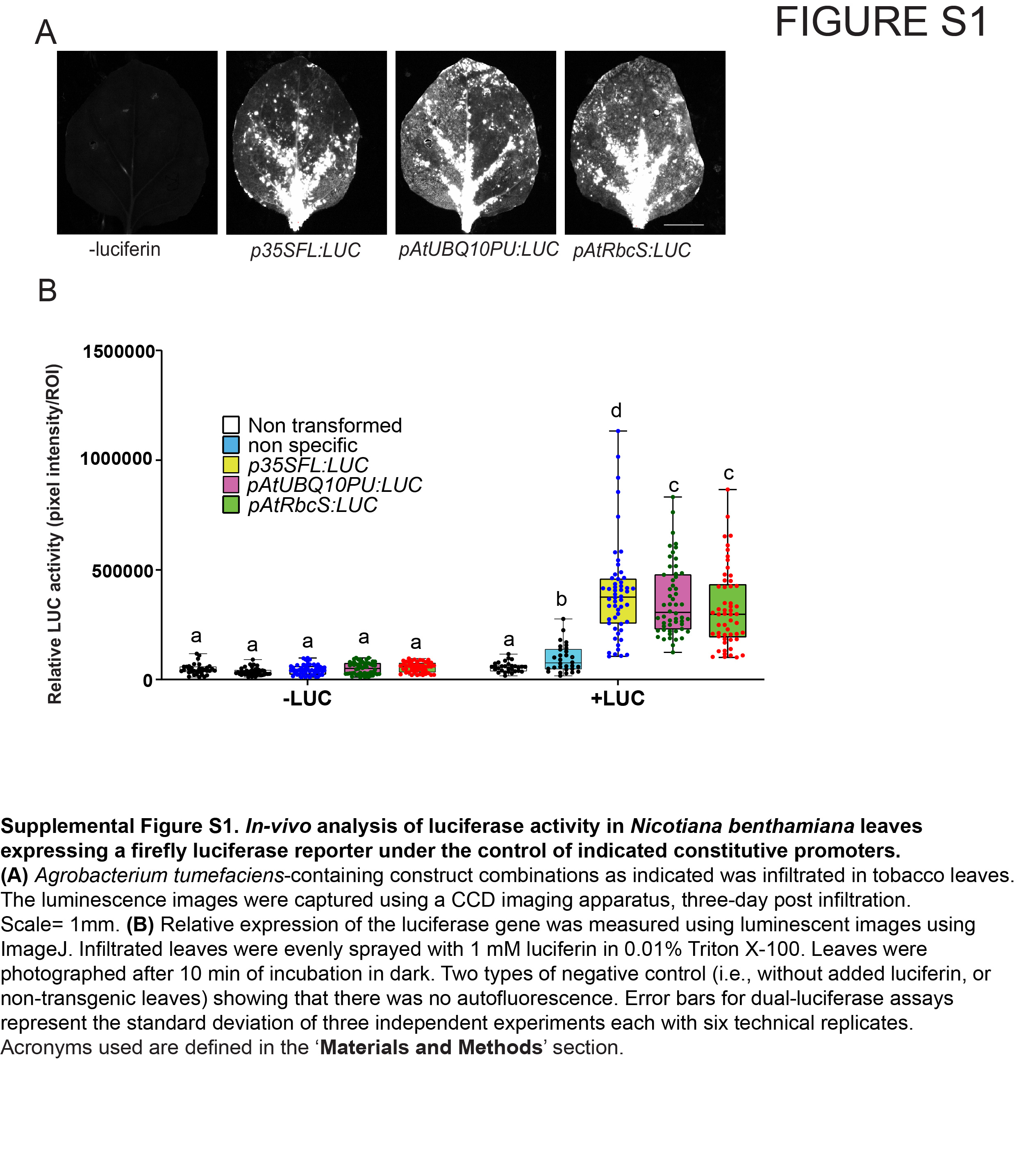

Supplement: Supplementary file 3 [file Image_1.JPEG]

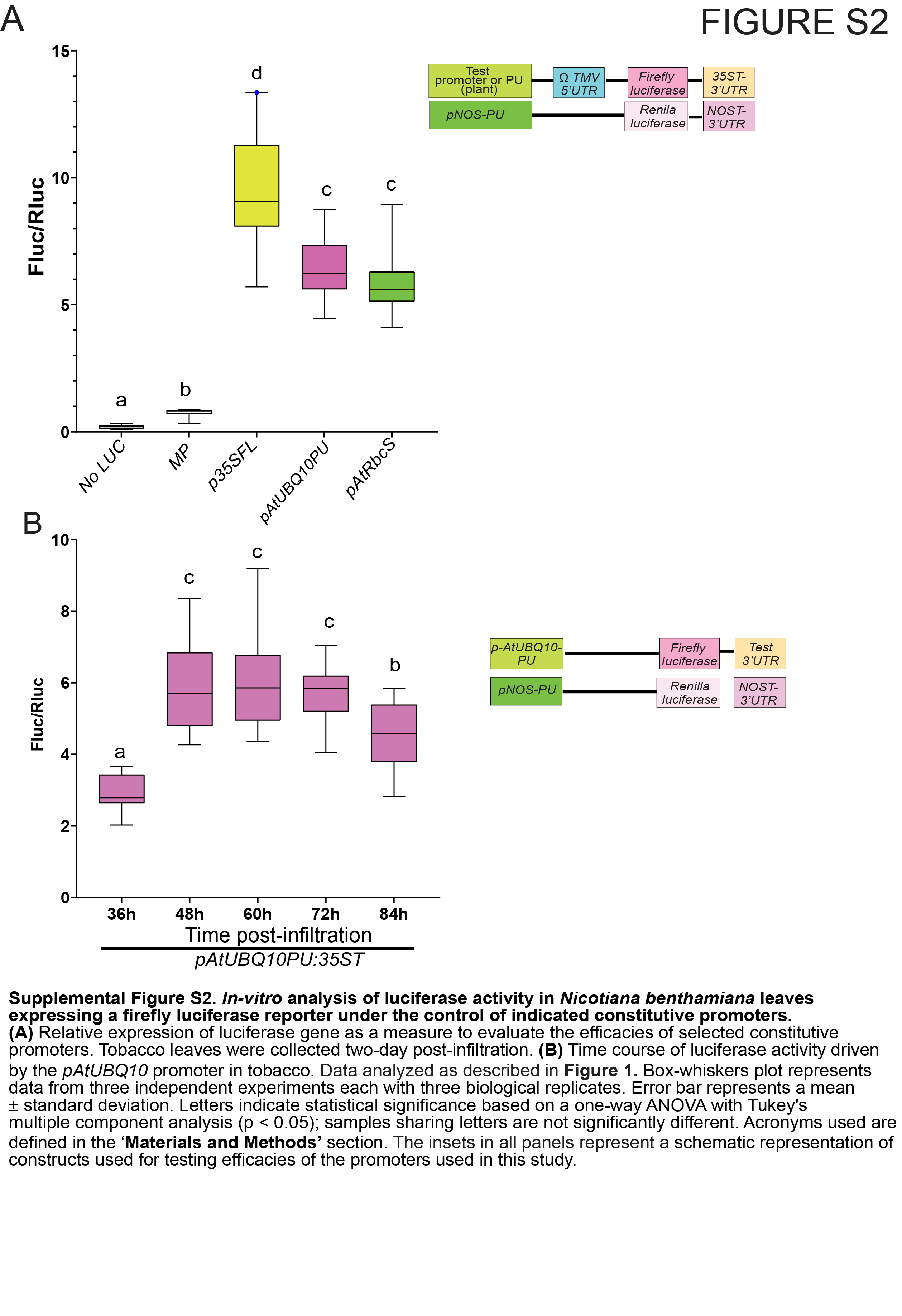

Supplement: Supplementary file 4 [file Image_2.JPEG]

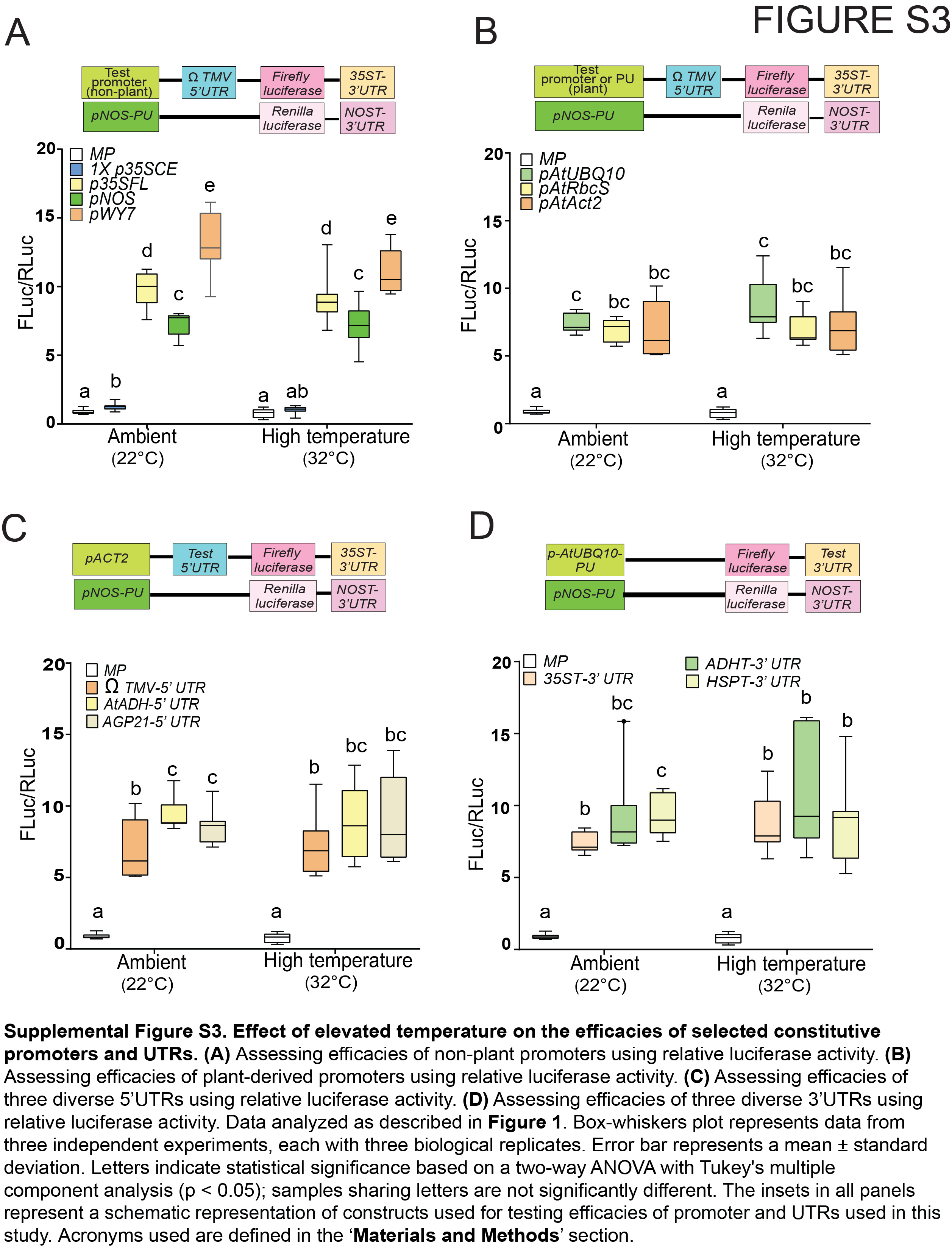

Supplement: Supplementary file 5 [file Image_3.JPEG]

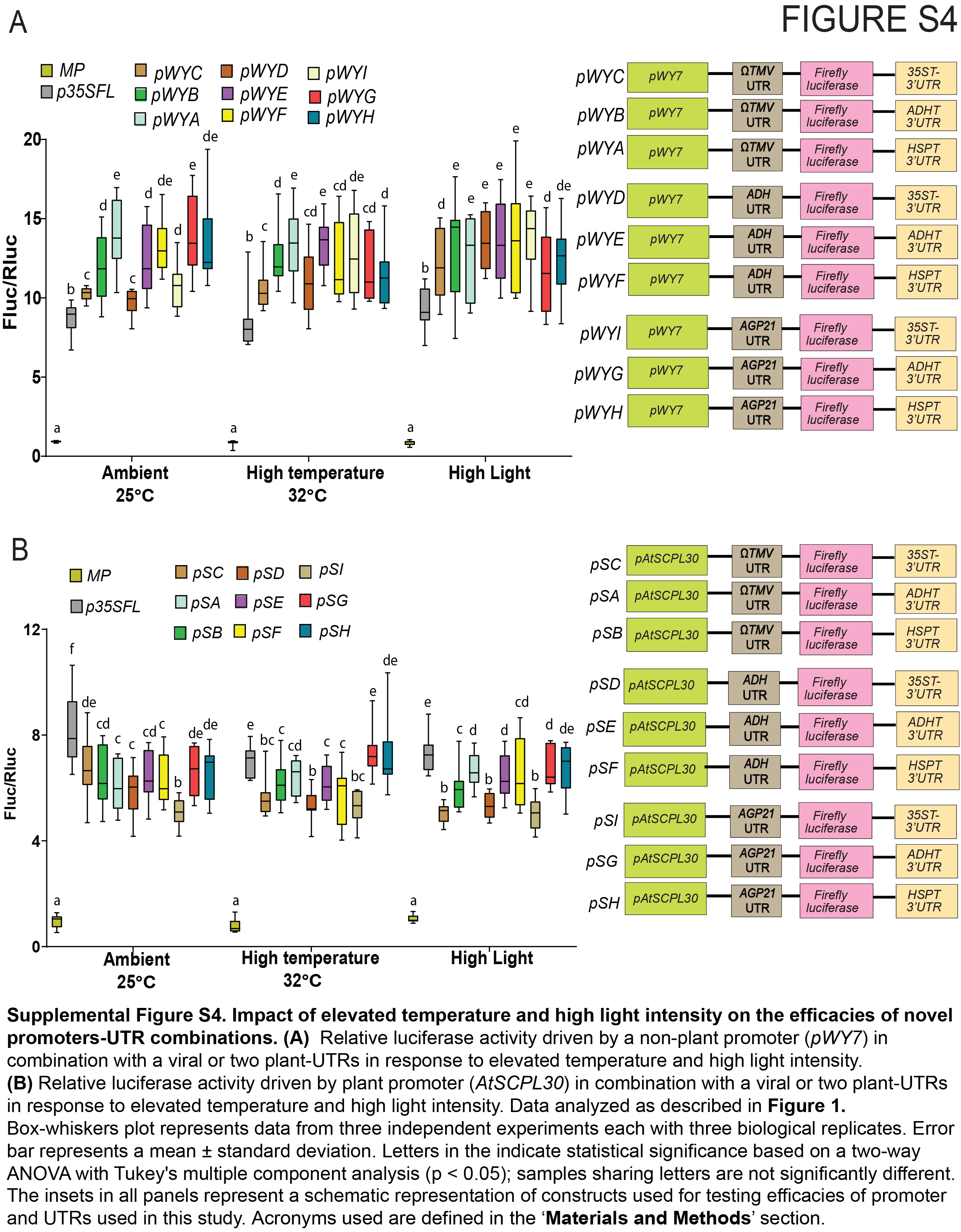

Supplement: Supplementary file 6 [file Image_4.JPEG]

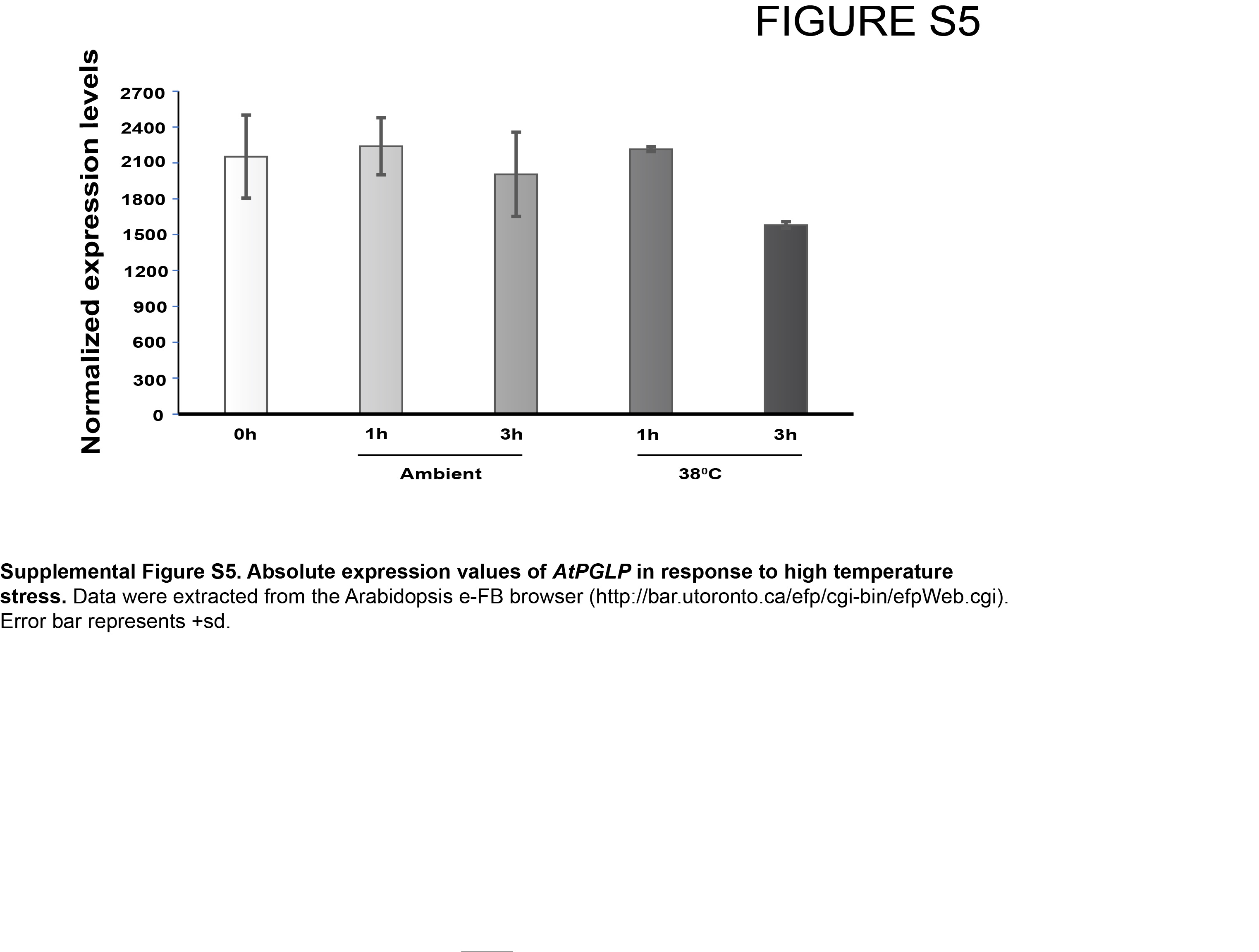

Supplement: Supplementary file 7 [file Image_5.JPEG]

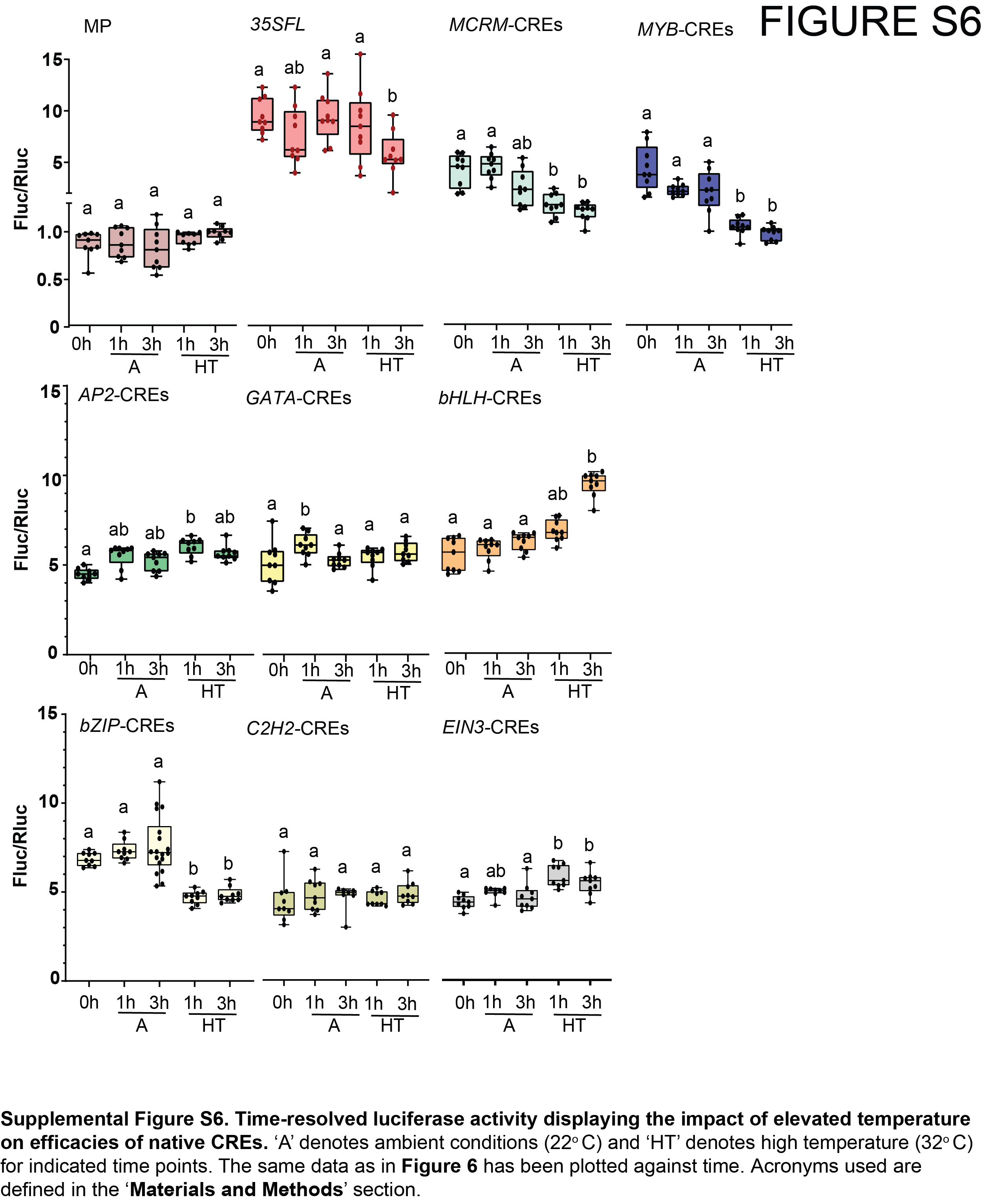

Supplement: Supplementary file 8 [file Image_6.JPEG]
